# Inhibitory Effects of New Epicatechin Oligomers on Nitric Oxide Production

**DOI:** 10.3390/ijms252011022

**Published:** 2024-10-14

**Authors:** Gyeong Han Jeong, Hanui Lee, Byung Yeoup Chung, Hyoung-Woo Bai

**Affiliations:** 1 Research Division for Biotechnology, Advanced Radiation Technology Institute (ARTI), Korea Atomic Energy Research Institute (KAERI), Jeongeup 56212, Republic of Korea; 2 Center for Companion Animal New Drug Development, Korea Institute of Toxicology (KIT), Jeongeup 56212, Republic of Korea; 3 Radiation Biotechnology and Applied Radioisotope Science, University of Science and Technology (UST), Daejeon 34113, Republic of Korea

**Keywords:** (–)-epicatechin, anti-inflammatory activity, nitric oxide production, oligomerization, procyanidin

## Abstract

The primary aim of this research was to identify the structural characteristics of three newly derived procyanidins from cold plasma-treated (–)-epicatechin, known for their anti-inflammatory properties. The newly generated compounds were isolated through column chromatography, and their chemical structures were elucidated through spectroscopic data analyses, including both one-dimensional and two-dimensional nuclear magnetic resonance (NMR) and mass spectrometry (MS) techniques. Furthermore, their absolute configurations were determined via circular dichroism (CD) spectroscopy. The inhibitory activity of the isolated compounds on nitric oxide (NO) production and expression levels of inducible NO synthase (iNOS) in lipopolysaccharide (LPS)-induced RAW 264.7 macrophages was evaluated. Three new procyanidins—methylenetrisepicatechin (**2**), isomethylenetrisepicatechin (**3**), and methylenebisepicatechin (**4**)—along with two reported dimeric flavan-3-ols (**5** and **6**), were identified from plasma-treated (–)-epicatechin (**1**). The unique oligomerized products **2** and **3** linked by methylene bridges significantly suppressed both NO production and iNOS expression, demonstrating higher anti-inflammatory activities in LPS-stimulated RAW 264.7 cells compared with the parent compound. The newly oligomerized procyanidins have potential applications in the treatment of inflammatory diseases owing to their significant anti-inflammatory properties.

## 1. Introduction

Inflammation serves as an essential immune response, providing a primary defense against tissue injury, infections, and various stimuli [1]. Nitric oxide (NO), produced by inducible NO synthase (iNOS), plays a critical role in regulating inflammation. Under normal physiological conditions, NO typically demonstrates anti-inflammatory effects [2]. However, excessive NO production, particularly by iNOS during an inflammatory response, can act as an inflammatory mediator [2,3]. This overabundance of NO can either interact with reactive oxygen species (ROS), leading to oxidative stress, or activate inflammatory cells, thereby exacerbating tissue damage. Therefore, maintaining appropriate NO levels and iNOS activity is essential for regulating inflammation and preventing tissue damage [4]. The intricate relationship between NO, iNOS, and inflammation has a significant influence on the development and progression of various diseases.

(–)-Epicatechin (**1**), a type of flavan-3-ol found in various plants, possesses two benzene rings and a dihydropyran structure with hydroxyl groups on the A and B rings and exists in both monomeric and oligomeric forms, the latter of which are known as procyanidins [5]. It is abundant in natural sources such as tea (*Camellia sinensis*), cocoa (*Theobroma cacao*), fruits (apples, grapes, berries, and cherries), red wine, and certain nuts, such as hazelnuts and pecans [6]. (–)-Epicatechin exhibits noteworthy functional properties, including antioxidant and anti-inflammatory activities, cardiovascular benefits, neuroprotective effects, improved metabolic functions, and potential anti-cancer properties [7]. Such health benefits make epicatechin a valuable compound in nutrition and therapeutics.

However, synthetic compounds have several significant drawbacks that must be considered in research and applications. The synthesis process often involves chemicals and methodologies that are harmful to the environment, potentially generating toxic by-products as waste [8]. Additionally, the development and production of synthetic compounds are both cost-intensive and time-consuming, posing economic and temporal challenges [9]. To overcome these obstacles, many researchers are proposing eco-friendly synthetic methods. Among these, cold plasma treatment technology stands out as a promising alternative due to its ability to induce significant chemical modifications without relying on hazardous solvents or high-energy input [10]. For example, plasma generates reactive oxygen species, free radicals, ultraviolet (UV) radiation, and ozone, which not only exhibit sterilizing effects but also induce changes in the major chemical components of natural products [10]. Previous studies utilizing cold plasma treatment technology have reported the formation of oligomers through methylene bridges in phenolic derivatives, resulting in compounds with higher antidiabetic activity than the parent compound [11,12]. This technology, by leveraging plasma’s high reactivity under ambient conditions, facilitates the selective modification of molecular structures, allowing for the generation of bioactive compounds with enhanced properties [12]. While there have been various studies, a comprehensive understanding of the mechanistic aspects and fundamentals of cold plasma treatment remains elusive, especially concerning the irradiation of naturally occurring secondary metabolites with diverse structural frameworks. Further exploration in this area holds promise for unveiling novel applications and insights into the potential of cold plasma technology. Continuing our efforts to develop bioactive agents from commonly found natural compounds, we describe a novel approach to the structural condensation of (–)-epicatechin through cold plasma modification. Our findings demonstrate that this process leads to the formation of unique procyanidin derivatives, which exhibit notably enhanced bioactivity, as evidenced by in vitro assays assessing NO production and iNOS expression in RAW 264.7 macrophages induced by lipopolysaccharide (LPS). Compared to the original (–)-epicatechin, these newly formed oligomers present significantly increased anti-inflammatory properties.

## 2. Results and Discussion

### 2.1. Isolation and Characterization of New Procyanidins

Cold plasma irradiation was conducted as previously described [13]. Briefly, a solution of (−)-epicatechin dissolved in methanol was subjected to cold plasma exposure for a duration of 60 min. Following this, the resulting compounds were analyzed via reversed-phase high performance liquid chromatography (HPLC). Through careful chromatographic separation, we successfully isolated three novel methylene-bridged derivatives (**2**−**4**) of (−)-epicatechin, in addition to two previously identified dimeric epicatechins: *bis*(8,8′-epicatechinyl)methane (**5**) and fangchengbisflavan A (**6**) (Figure S1).

**Figure 1:**
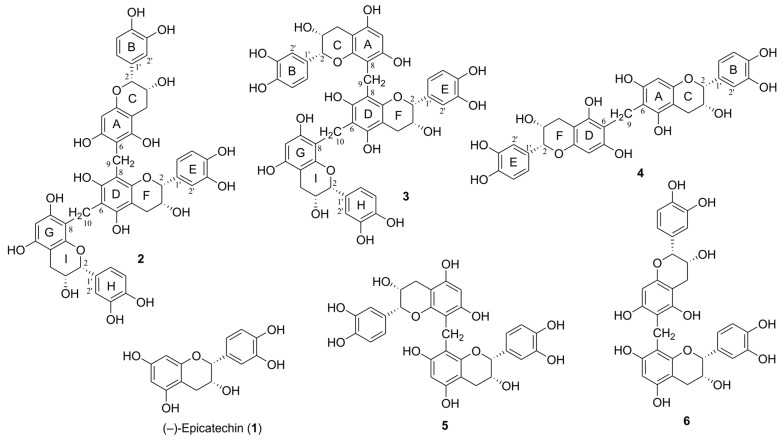
Structures of oligomerized products **2**–**6** of (–)-epicatechin (**1**). **2**: methylenetrisepicatechin [epicatechin (6→8) epicatechin (6→8) epicatechin], **3**: isomethylenetrisepicatechin [epicatechin (8→8) epicatechin (6→8) epicatechin], **4**: methylenebisepicatechin [epicatechin (6→6) epicatechin], **5**: *bis*(8,8′-epicatechinyl)methane [epicatechin (8→8) epicatechin], **6**: fangchengbisflavan A [epicatechin (6→8) epicatechin].

**Figure 2:**
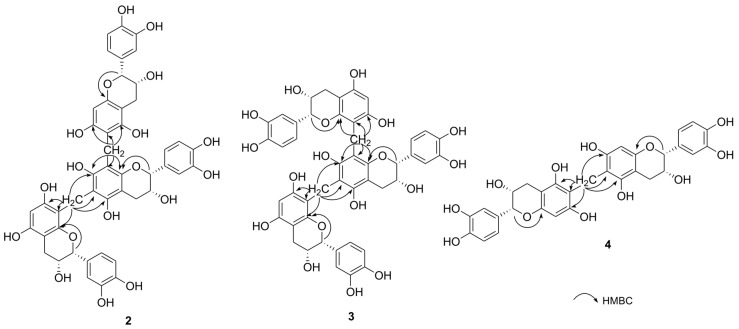
Key HMBC correlations of **2**–**4**.

**Figure 3:**
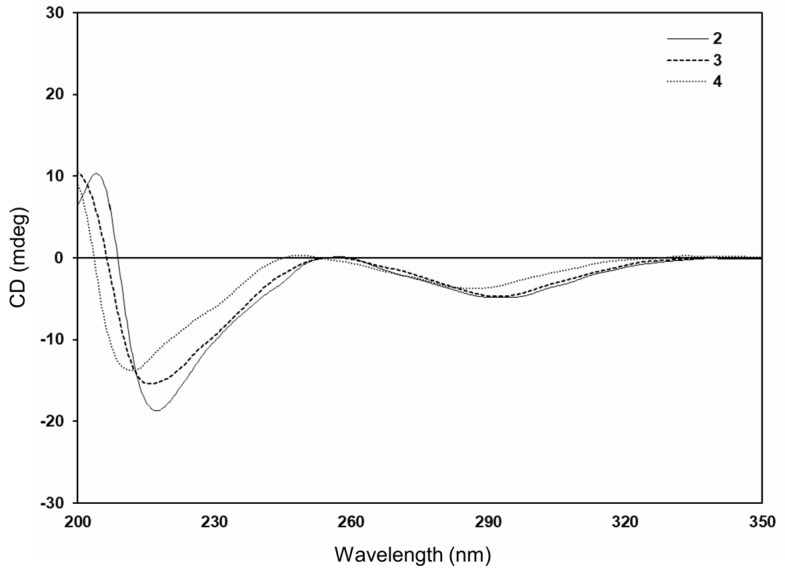
CD spectra of **2**–**4**.

Compound **2** was isolated as a brown amorphous powder with optical activity observed at [α]^20^_D_ −145.9 (*c* 0.1, MeOH). The high-resolution fast atom bombardment (HRFAB) mass spectrum in the positive mode displayed a molecular ion peak at *m/z* 894.2359 [M]^+^, corresponding to a molecular formula of C_47_H_42_O_18_. The UV spectrum showed maximum absorption at 205 nm, with an additional broad band around 281 nm, indicating the presence of a flavan 3-ols skeleton [14]. The ^1^H NMR spectrum of **2** (Table 1) displayed three sets of ABX-type aromatic protons at *δ*_H_ 7.05 (1H, d, *J* = 1.8 Hz, H-2′H), 6.96 (1H, br s, H-2′B), 6.86 (1H, br s, H-2′E), 6.85 (1H, dd, *J* = 7.8, 1.8 Hz, H-6′H), 6.72 (1H, br s, H-6′B), 6.78 (1H, d, *J* = 7.8 Hz, H-5′H), 6.70 (1H, br s, H-5′E), 6.69 (1H, d, *J* = 8.4 Hz, H-5′B), and 6.65 (1H, dd, *J* = 8.4, 1.8 Hz, H-6′B). Additionally, three sets of oxygenated methine protons with small-coupling constants (*J*_2,3_) were observed at *δ*_H_ 4.91 (1H, br s, H-2I), 4.71 (1H, br s, H-2C), 4.57 (1H, br s, H-2F), 4.19 (1H, m, H-3I), 4.10 (1H, m, H-2F), and 4.05 (1H, m, H-3C). In addition, three sets of methylene protons appeared at *δ*_H_ 2.86 (1H, dd, *J* = 16.8, 4.2 Hz, H-4I), 2.79 (1H, dd, *J* = 16.8, 3.0 Hz, H-4I), 2.78 (1H, dd, *J* = 16.8, 4.2 Hz, H-4C), 2.68 (1H, dd, *J* = 16.8, 4.2 Hz, H-4F), 2.62 (1H, dd, *J* = 16.8, 3.0 Hz, H-4C), and 2.60 (1H, dd, *J* = 16.8, 3.0 Hz, H-4F). Furthermore, two isolated aromatic singlets were identified at *δ*_H_ 6.03 (1H, s, H-6G) and 5.93 (1H, s, H-8A), indicating the presence of three epicatechin moieties (Figure 1) [15]. The ^13^C and heteronuclear single quantum coherence (HSQC) NMR spectra of **2** confirmed the presence of trimeric (–)-epicatechin units with resonances at *δ*_C_ 81.2 (C-2I), 80.3 (C-2C), 80.1 (C-2F), 67.2 (C-3C), 66.9 (C-3F), 66.8 (C-3I), 29.3 (C-4C), 29.2 (C-4F), and 29.1 (C-4I). Additionally, two *geminally* coupled methylene protons were observed at *δ*_H_ 3.96/3.83 (d, *J* = 15.6 Hz, *δ*_C_ 17.7) and 3.79/3.72 (d, *J* = 15.6 Hz, *δ*_C_ 17.1). The positions of the two methylene bridges within the epicatechin trimer were identified by analyzing key correlations in the heteronuclear multiple bond correlation (HMBC) spectrum, which revealed connections between C-6A and C-8D, as well as C-6D and C-8G (Figure 2). The absolute configurations at C-2 and C-3 were established by comparing the circular dichroism (CD) spectrum to similar natural flavan 3-ols [16]. The CD spectra showed negative Cotton effects at 216 and 287 nm, consistent with a 2*R*, 3*R*-configuration of the structural units (Figure 3). Thus, the absolute structure of compound **2** was elucidated as methylenetrisepicatechin, as shown in Figure 1.

The HRFAB-MS spectrum of compound **3** showed a molecular ion peak at *m/z* 894.2372 [M]^+^, which corresponds to the molecular formula C_47_H_42_O_18_, matching that of compound **2** (Table 1). The 1D NMR and UV spectra of compound **3** were found to be almost identical to those of compound **2**, indicating that these two substances are structural isomers (Figure 1). Key correlations in the HMBC spectrum of **3** indicated that its structure is a trimeric epicatechin derivative linked through a methylene bridge between the C-8A and C-8D positions and the C-6D and C-8G positions (Figure 2). The same 2*R*, 3*R*-configuration as in **3** was inferred from the diagnostic small coupling constants (*J*_2,3_) and negative Cotton effects at approximately 286 nm in its CD spectrum (Figure 3) [16,17]. Based on this evidence, the trimeric (−)-epicatechin derivative in new compound **3** was assigned as isomethylenetrisepicatechin (Figure 1).

Compound **4** was purified as a brown amorphous powder, with [α]^20^_D_ −37.9 (*c* 0.1, MeOH). Its molecular formula, determined to be C_31_H_28_O_12_ based on HRFAB-MS and ^13^C NMR spectroscopic data, corresponds to dimeric epicatechin. The ^1^H NMR spectrum of **4** (Table 1) revealed an ABX-type aromatic system at *δ*_H_ 6.74 (2H, d, *J* = 2.4 Hz, H-2′B, 2′E), 6.77 (2H, dd, *J* = 7.8, 2.4 Hz, H-6′B, 6′E), and 6.74 (2H, d, *J* = 2.4 Hz, H-5′B, 5′E). Two oxygenated methine signals appeared at *δ*_H_ 4.78 (2H, br s, H-2C, 2F) and 4.15 (2H, m, H-3C, 3F) with small coupling constants, indicative of a 2,3-*cis* configuration [17]. In the ^1^H NMR spectrum, a methylene group appeared at *δ*_H_ 2.88 (2H, dd, *J* = 16.8, 4.8 Hz, H-4C, 4F) and 2.75 (2H, dd, *J* = 16.8, 3.0 Hz, H-4C, 4F). Additionally, there was a single methylene proton observed at *δ*_H_ 3.74 (2H, s, H-9) as well as an isolated aromatic proton at *δ*_H_ 6.04 (2H, s, H-8A, 8D). Upon comparing the 1D NMR and MS spectral data for compound **4** with that of previously reported compounds showing symmetrical structures like *bis*(8,8′-epicatechinyl)methane (**5**), it appears that these two dimeric products are structural isomers [18]. The difference between dimeric epicatechin derivatives **4** and **5** lies in the linkage points, which are either at C-6A and C-6D or C-8A and C-8D. The position of the methylene linkage in **4** was confirmed to be C-6A and C-6D using key HMBC correlations (Figure 2). The absolute configuration at C-2 and C-3 in **4** was determined by CD spectral analysis [16]. The CD measurements showed a negative Cotton effect at 284 nm, indicative of a 2*R*, 3*R*-configuration of the upper and lower units (Figure 3). As a result, the newly identified symmetrical structure of compound **4** was assigned as methylenebisepicatechin (Figure 1).

The previously reported dimeric epicatechin derivatives, compounds **5** and **6**, were identified as *bis*(8,8′-epicatechinyl)methane and fangchengbisflavan A, respectively, by comparing their spectroscopic data (1D and 2D NMR, MS) with the literature values. These dimeric epicatechins were first isolated and identified from the pericarps of *Litchi chinensis* and the leaves of *Camellia fangchengensis*, respectively [18,19].

The potential to oligomerize (−)-epicatechin into novel structures using cold plasma treatment presents significant implications for the field of natural product chemistry and pharmacognosy. The formation of three new methylene-bridged derivatives (**2**–**4**), in addition to two known dimeric epicatechin derivatives (**5** and **6**), highlights the versatility of cold plasma as a tool for molecular modification (Figure S38). Cold plasma treatment offers a green and efficient alternative to traditional chemical synthetic methods, reducing the need for harsh reagents and extensive purification. The structural elucidation of the oligomeric compounds presented herein expands our understanding of the chemical diversity and potential bioactivity of procyanidins. Future research should focus on exploring the biological activities of these newly synthesized oligomers. For example, given the known antioxidant properties of (−)-epicatechin and its derivatives, these new compounds may exhibit unique pharmacological profiles worthy of investigation. Additionally, the methodology employed in this study could be applied to other flavonoids and natural products, paving the way for the discovery of novel compounds with potential therapeutic benefits. The development of scalable cold plasma treatment techniques can further enhance the practical applications of this technology in both research and industry. As a proof of concept, the newly synthesized methylene-bridged derivatives of (–)-epicatechin were tested for their capability of inhibiting NO production and iNOS expression.

### 2.2. Inhibitory Activities on NO Production and iNOS Expression

**Figure 4:**
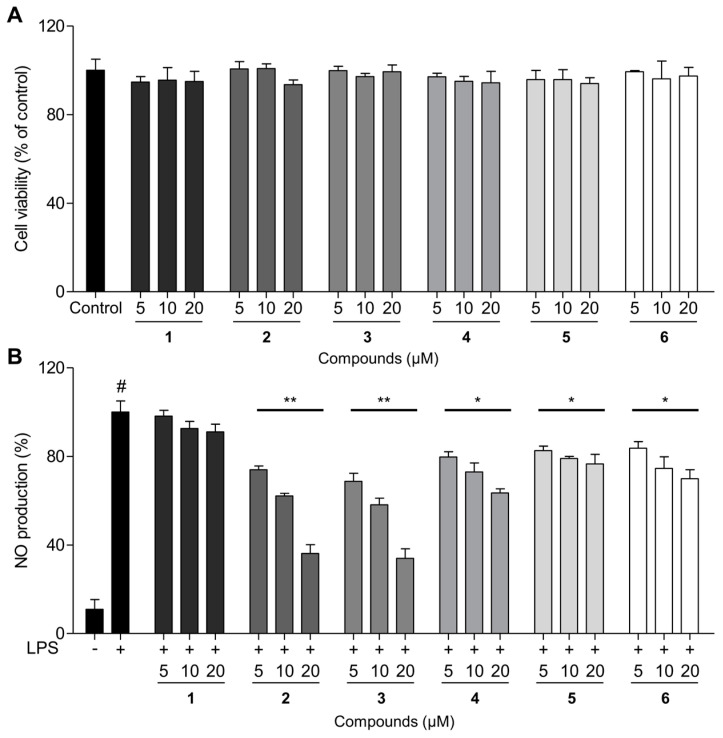
Effects of the epicatechin oligomers **2–6** on LPS-induced in RAW 264.7 cells. (**A**) MTT assay results for cell viability in RAW 264.7 cells following treatment with isolated compounds (5, 10, and 20 µM) over a 24 h period. (**B**) The cells were pretreated with the isolated compounds at concentrations of 5, 10, and 20 µM for 1 h and then exposed to LPS (0.1 µg/mL) for 24 h. NO levels in the culture medium were measured. Untreated cells without isolated compounds or LPS exposure served as the control group. The results are expressed as mean ± SD (*n* = 3). ^#^ *p* < 0.05 represents significant differences compared to the control group. * *p* < 0.05 and ** *p* < 0.01 represent significant differences compared to only the LPS-induced group. **1**: (–)-epicatechin, **2**: methylenetrisepicatechin, **3**: isomethylenetrisepicatechin, **4**: methylenebisepicatechin, **5**: *bis* 8,8′-epicatechinylmethane, **6**: fangchengbisflavan A.

Excessive NO production can lead to inflammation and tissue damage, contributing to various inflammatory diseases [2]. By reducing NO levels, these compounds may help mitigate inflammation and promote better overall health [4]. The NO production inhibitory effects of the isolated procyanidins were examined in RAW 264.7 macrophages using the Griess reagent method [20]. To determine cytotoxicity, a cell viability assay was conducted on RAW 264.7 cells at concentrations of 5, 10, and 20 μM. The results showed no cytotoxic effects for any of the compounds, even at the highest concentration of 20 μM, as confirmed by the cell viability data (Figure 4A).

**Figure 5:**
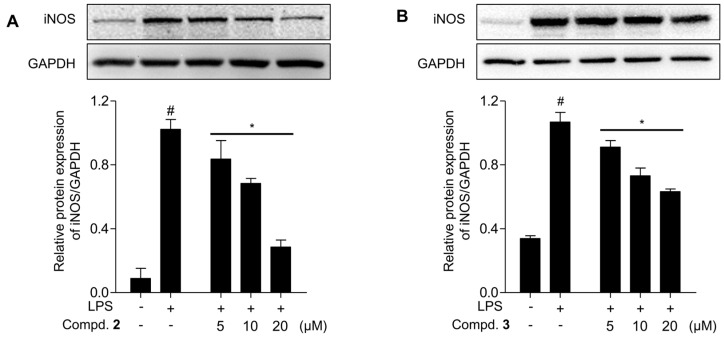
Effects of trimerization products **2** and **3** on iNOS protein expression in LPS-induced RAW 264.7 cells. The cells were pretreated with compounds **2** and **3** (5, 10, and 20 µM) for 1 h and then incubated with LPS (0.1 µg/mL) for 24 h. iNOS protein levels were determined by immunoblotting, and GAPDH was used as the internal control. The results are expressed as mean ± SD (*n* = 3). ^#^ *p* < 0.05 represents significant differences compared to the control group. * *p* < 0.05 represents significant differences compared to only the LPS-induced group. (**A**) methylenetrisepicatechin, (**B**) isomethylenetrisepicatechin.

Figure 4B shows that 20 μM solutions of trimerized epicatechin compounds **2** and **3** linked by methylene bridges significantly inhibited NO production by 63.8 ± 2.5% and 66.0 ± 3.4%, respectively, in the absence of cytotoxicity when compared with LPS-treated groups. In contrast, the dimerized products **4**–**6** exhibited lower inhibition of NO production in macrophages. The enhanced inhibition of NO production by compounds **2** and **3** highlights the structural advantage of trimeric epicatechin derivatives over dimeric ones, suggesting that the methylene bridge connection may contribute to increased bioactivity. Additionally, the absence of cytotoxicity up to 20 μM further supports the potential of these compounds as safe therapeutic agents for inflammation-related conditions. To explore whether the new oligomerized analogs **2** and **3** exert their inhibitory effects on NO production by modulating iNOS expression, we examined the iNOS protein levels using western blots (Figure 5). LPS treatment elevated iNOS expression in RAW 264.7, but methylenetrisepicatechin (**2**) and isomethylenetrisepicatechin (**3**) significantly suppressed iNOS protein expression, which is involved in NO production. These findings suggested that the newly oligomerized products **2** and **3** inhibited NO production by downregulating iNOS protein expression.

The inhibitory activity of compounds **2** and **3** on iNOS expression is particularly significant, as iNOS is a key enzyme responsible for sustained NO production during inflammatory responses. The suppression of iNOS expression by these compounds indicates a potential mechanism by which they reduce NO production, highlighting their role as effective anti-inflammatory agents. Furthermore, this downregulation of iNOS could help mitigate chronic inflammation, as prolonged iNOS activation is often associated with various inflammatory diseases.

The findings of this study reveal significant insight into the potential therapeutic applications of (−)-epicatechin oligomers. The ability of compounds **2** and **3** to inhibit NO production and suppress iNOS protein expression without inducing cytotoxic effects highlights their potential use as anti-inflammatory agents. These results suggest that methylene-bridged procyanidins could be developed into effective treatments for inflammatory diseases in which excessive NO production plays a critical role. Additionally, the potent effects of these trimeric compounds on both NO production and iNOS expression suggest that they may be more efficient than other flavonoid-based treatments. Future research should investigate their potential to provide a more targeted approach to reducing inflammation by specifically modulating iNOS activity.

The methodology employed in this research, that is, utilizing cold plasma treatment for the oligomerization of (−)-epicatechin, presents a novel and environmentally friendly approach to synthesizing complex natural product derivatives. This technique can be adapted to modify other flavonoids and bioactive compounds, thereby broadening the scope of natural product chemistry. Furthermore, cold plasma technology has proven to be a versatile tool not only for generating novel bioactive compounds but also for offering a sustainable and green chemistry approach. This technique could be extended to other natural compounds, facilitating the discovery of new drugs with fewer environmental impacts than traditional synthetic methods. Future studies should focus on the in vivo efficacy and safety of these compounds and explore their mechanisms of action in greater detail. Additionally, investigating the potential synergistic effects of these oligomers with other anti-inflammatory agents may provide valuable insight into combinational therapies for complex inflammatory conditions.

## 3. Materials and Methods

### 3.1. Chemicals and Instruments

The (–)-epicatechin used in this study was sourced from Sigma-Aldrich (St. Louis, MO, USA). All other reagents were of analytical grade. UV spectral data were collected with a T-60 spectrophotometer (PG Instruments, Leicestershire, UK), while circular dichroism (CD) spectra and optical rotation measurements were obtained using a JASCO J-1500 spectrometer and a JASCO P-2000 polarimeter (JASCO, Tokyo, Japan). The ^1^H and ^13^C NMR spectra were recorded on a Bruker Avance NEO-600 instrument (Bruker, Karlsruhe, Germany) at 600 and 150 MHz, respectively. Chemical shifts are reported in *δ* (ppm), with CD_3_OD (*δ*_H_ 3.35; *δ*_C_ 49.8) as the solvent reference, relative to tetramethylsilane (TMS, 0.0 ppm). Standard pulse sequences were used for all 2D NMR measurements, with a *J*_HZ_ value of 8 Hz for HMBC spectra. Fast atom bombardment mass spectrometry (FAB-MS) was conducted on a JMS-700 spectrometer (JEOL, Tokyo, Japan). Column chromatography utilized YMC GEL ODS AQ 120-50S columns (particle size 50 μm; YMC Co., Kyoto, Japan), while analytical reversed-phase HPLC was performed on a Shimadzu LC-20AD (Shimadzu, Tokyo, Japan) with a YMC-Pack ODS A-302 column (4.6 mm i.d. × 150 mm, particle size 5 μm; YMC Co., Kyoto, Japan) employing an elution gradient of MeCN in 0.1% HCOOH at a flow rate of 1.0 mL/min, detected by a Shimadzu SPD-M20A photodiode array detector (Shimadzu, Tokyo, Japan).

### 3.2. Sample Preparation and Isolation Procedure

The plasma treatment setup included a process chamber outfitted with a Dielectric Barrier Discharge (DBD) system and an accompanying power supply unit [13]. The chamber, made of Teflon due to its low chemical reactivity, has dimensions of 150 × 150 × 275 (h) mm³. The DBD apparatus comprised four surface DBD sources, each constructed from a 0.6 mm thick fused silica plate measuring 100 × 100 mm² and two nickel–chromium alloy metal sheet electrodes. One electrode featured 6 × 6 open-surface patterns, each being a rounded square of 9 × 9 mm², whereas the other electrode was solid with no open areas. The power supply included an arbitrary waveform generator (Tektronix AFG3021C) and a high-voltage power amplifier (Trek 5/80). During operation, a sinusoidal waveform with a frequency of 2.5 kHz and a peak-to-peak voltage of 4 kV was applied between the electrodes, generating surface discharge across the open patterns in ambient air without an additional gas supply. Power dissipation by the plasma was measured using a high-voltage probe (Tektronix P6015A), a 10:1 voltage probe (Tektronix P2100), and a 100 nF capacitor. The power dissipated by the plasma was 65 W (±5%), with room temperature consistently maintained during the entire process. The chamber temperature was tracked using a digital hygrometer, both before and after the treatment of the sample.

A solution of (–)-epicatechin (400 mg) in methanol (400 mL) was treated with DBD plasma for 60 min. The resulting compounds were analyzed by reversed-phase HPLC. Post-treatment, the dried solution was dissolved in 10% MeOH (100 mL) and then partitioned with ethyl acetate (3 × 100 mL), yielding an ethyl acetate-soluble fraction weighing 311.0 mg. A portion of this ethyl acetate extract (303 mg) underwent column chromatography on a YMC GEL ODS AQ 120-50S column (1 cm i.d. × 39 cm) with an aqueous methanol solvent. This process isolated pure procyanidins **2** (14.4 mg, *t*_R_ 14.4 min), **3** (3.8 mg, *t*_R_ 16.0 min), **4** (5.7 mg, *t*_R_ 15.9 min), **5** (25.9 mg, *t*_R_ 12.8 min), **6** (68.3 mg, *t*_R_ 14.6 min), and the original material (–)-epicatechin (**1**, 58.3 mg, *t*_R_ 9.9 min) (Figure S2).

Methylenetrisepicatechin (**2**): brown amorphous powder, [α]^20^_D_ −145.9 (*c* 0.1, MeOH); UV λ_max_ MeOH nm (log ε): 205 (3.75), 281 (1.16) nm; CD (MeOH) Δε (nm): 217 (−18.7), 293 (−4.8) nm; ^1^H and ^13^C NMR, see Table 1; FAB-MS *m/z* 894 [M]^+^, HRFAB-MS *m/z* 894.2359 [M]^+^ (calculated for C_47_H_42_O_18_, 894.2371) (Figures S3–S9, S37).

Isomethylenetrisepicatechin (**3**): brown amorphous powder, [α]^20^_D_ −11.5 (*c* 0.1, MeOH); UV λ_max_ MeOH nm (log ε): 204 (4.05), 281 (1.29) nm; CD (MeOH) Δε (nm): 215 (−15.4), 292 (−4.7) nm; ^1^H and ^13^C NMR, see Table 1; FAB-MS *m/z* 894 [M]^+^, HRFAB-MS *m/z* 894.2352 [M]^+^ (calculated for C_47_H_42_O_18_, 894.2371) (Figures S10–S15, S37).

Methylenebisepicatechin (**4**): brown amorphous powder, [α]^20^_D_ −37.9 (*c* 0.1, MeOH); UV λ_max_ MeOH nm (log ε): 205 (3.69), 287 (1.16) nm; CD (MeOH) Δε (nm): 212 (−13.7), 287 (−3.8) nm; ^1^H and ^13^C NMR, see Table 1; FAB-MS *m/z* 592 [M]^+^, HRFAB-MS *m/z* 592.1584 [M]^+^ (calculated for C_31_H_28_O_12_, 592.1581) (Figures S16–S22, S37).

*bis*(8,8′-Epicatechinyl)methane (**5**): brown amorphous powder, [α]^20^_D_ −193.2 (c 0.1, MeOH); ^1^H NMR (600 MHz, CD_3_OD): *δ* 6.95 (2H, br s, H-2′B, E), 6.73 (2H, br s, H-5′B, E), 6.72 (2H, br s, H-6′B, E), 5.95 (2H, s, H-6A, D), 4.76 (2H, br s, H-2C, F), 4.10 (2H, dd, *J* = 4.8, 3.6 Hz, H-3C, F), 3.88 (2H, s, H-9A, D), 2.82 (2H, dd, *J* = 16.2, 4.8 Hz, H-4C, F), 2.68 (2H, dd, *J* = 16.8, 3.0 Hz, H-4C, F); ^13^C NMR (CD_3_OD, 150 MHz): *δ* 155.9 (C-5A, D), 155.2 (C-7A, D), 153.7 (C-8aA, D), 145.9 (C-4′B, E), 145.8 (C-3′B, E), 131.7 (C-1′B, E), 119.7 (C-6′B, E), 116.0 (C-5′B, E), 115.4 (C-2′B, E), 106.7 (C-8A, D), 100.5 (C-4aA, D), 96.8 (C-6A, D), 80.4 (C-2C, F), 67.2 (C-3C, F), 29.0 (C-4C, F), 16.8 (C-9A, D); FAB-MS *m/z* 593 [M + H]^+^ (Figures S23–S29).

Fangchengbisflavan A (**6**): yellow amorphous powder, [α]^20^_D_ −88.9 (c 0.1, MeOH); ^1^H NMR (CD_3_OD, 600 MHz): *δ* 7.09 (1H, d, *J* = 1.8 Hz, H-2′E), 6.93 (1H, d, *J* = 1.2 Hz, H-2′B), 6.90 (1H, dd, *J* = 8.4, 1.8 Hz, H-6′E), 6.81 (1H, d, *J* = 8.4 Hz, H-5′E), 6.75 (1H, dd, *J* = 7.8, 1.2 Hz, H-6′B), 6.73 (1H, d, *J* = 7.8 Hz, H-5′B), 6.07 (1H, s, H-6D), 5.98 (1H, s, H-8A), 4.99 (1H, br s, H-2F), 4.73 (1H, br s, H-2C), 4.18 (1H, m, H-3F), 4.11 (1H, m, H-3C), 3.78 (1H, d, *J* = 15.6 Hz, H-9), 3.75 (1H, d, *J* = 15.6 Hz, H-9), 2.89 (1H, dd, *J* = 16.8, 4.8 Hz, H-4F), 2.79 (1H, dd, *J* = 16.8, 4.8 Hz, H-4C), 2.78 (1H, dd, *J* = 16.8, 3.0 Hz, H-4F), 2.69 (1H, dd, *J* = 16.8, 3.0 Hz, H-4C); ^13^C NMR (CD_3_OD, 150 MHz): *δ* 156.2 (C-8aD), 155.4 (C-8aD), 155.3 (C-7A), 154.7 (C-7D), 154.3 (C-5A), 153.1 (C-5D), 146.3 (C-3′E), 146.1 (C-3′B), 145.9 (C-4′E), 145.7 (C-4′B), 132.2 (C-1′E), 131.0 (C-1′B), 120.0 (C-6′E), 119.4 (C-6′B), 116.1 (C-5′E), 115.9 (C-5′B), 115.6 (C-2′B), 115.3 (C-2′E), 108.1 (C-8D), 106.7 (C-6A), 101.4 (C-4aD), 100.8 (C-4aA), 97.2 (C-6D), 96.5 (C-8A), 81.4 (C-2E), 79.8 (C-2C), 67.5 (C-3E), 67.0 (C-3C), 29.6 (C-4E), 29.3 (C-4C), 17.4 (C-9); FAB-MS *m/z* 593 [M + H]^+^ (Figures S30–S36).

### 3.3. Cell Culture

The RAW 264.7 cell line used in this study was sourced from the Korean Cell Line Bank at Seoul National University (Seoul, Korea). The macrophages were maintained in a sterile environment at 37 °C with a humidified 5% CO_2_ atmosphere. The culture medium was Dulbecco’s Modified Eagle Medium (DMEM) from GIBCO Invitrogen Corp. (Carlsbad, CA, USA), which was supplemented with 10% fetal bovine serum (FBS) and 1% penicillin/streptomycin (GIBCO Invitrogen Corp.).

### 3.4. Cell Viability Assay

The MTT assay was employed to evaluate the cell viability effects of procyanidins **2**–**6**, which were derived from plasma-treated (–)-epicatechin (**1**), on RAW 264.7 cells [21]. Cells were seeded at a density of 5 × 10⁴ cells/well in 96-well plates and incubated for 24 h at 37 °C. They were then treated with (–)-epicatechin and compounds **2**–**6** at concentrations ranging from 5 to 20 μM and further incubated for another 24 h at 37 °C. MTT solution (0.5 mg/mL) was added, and the plates were incubated for an additional 3 h at 37 °C to facilitate formazan blue formation. Following the removal of the medium, formazan crystals were dissolved in dimethyl sulfoxide (DMSO), and cell viability was assessed by measuring absorbance at 570 nm with a spectrophotometer (Tecan, Männedorf, Switzerland). The control group exhibited 100% viability.

### 3.5. Measurement of Nitric Oxide Production

RAW 264.7 cells were plated in 96-well plates at a density of 5 × 10⁴ cells/well and incubated for 24 h at 37 °C. Following this, the cells were pre-treated with different concentrations of isolated compounds **2**–**6** for 2 h before being stimulated with LPS (0.1 μg/mL) for 24 h at 37 °C. Nitric oxide production was assessed by reacting the supernatant from the macrophage cultures with the Griess reagent (Sigma-Aldrich, St. Louis, MO, USA) based on a modified Griess method. To execute the assay, 100 μL of the supernatant was combined with 100 μL of the Griess reagent at room temperature and gently shaken for 20 min. The absorbance of the resulting reaction mixture was then measured at 548 nm using a microplate reader (Tecan, Männedorf, Switzerland) [20].

### 3.6. Western Blot Analysis

RAW 264.7 macrophage cells treated with the samples were collected and lysed using a radioimmunoprecipitation assay (RIPA) buffer (Rockland Immunochemicals Inc., Limerick, PA, USA). Following lysis, cell debris was removed via centrifugation, and the protein concentration was measured using a bicinchoninic acid (BCA) protein assay kit (Thermo Fisher Scientific, Inc., Waltham, MA, USA) in accordance with the manufacturer’s instructions. Equal protein amounts (30 μg) from each lysate were separated by 10% SDS-PAGE and transferred to polyvinylidene difluoride (PVDF) membranes. These membranes were blocked with 5% nonfat milk in Tris-buffered saline with Tween-20 for 1 h at room temperature. They were then incubated overnight at 4 °C with primary antibodies against iNOS (cat. #2977) and GAPDH (cat. #2118), both diluted at 1:1000 (antibodies from Cell Signaling Technology, Danvers, MA, USA). Subsequently, the blots were treated with horseradish peroxidase (HRP)-conjugated anti-rabbit IgG (cat. #7074, diluted 1:3000; Cell Signaling Technology) for 2 h at room temperature, and protein bands were visualized using a chemiluminescent substrate (Figure S39).

### 3.7. Statistical Analysis

Each experiment was performed at least three times, and the results were expressed as the mean ± SD. For multiple comparisons, one-way analysis of variance (ANOVA) was used, followed by Tukey’s multiple comparison test. *p* ≤ 0.05 was considered to indicate a statistically significant result.

## 4. Conclusions

We have confirmed that (–)-epicatechin could be oligomerized in a straightforward manner, providing three new compounds (**2**–**4**), along with two known compounds (**5** and **6**). Novel (–)-epicatechin trimers **2** and **3** demonstrated potent anti-inflammatory activities, specifically inhibiting NO production and iNOS expression in LPS-induced RAW 264.7 macrophages, compared with the original (–)-epicatechin. The significant inhibition of NO production and iNOS expression by these trimeric derivatives underscores their potential as effective anti-inflammatory agents, which could offer therapeutic benefits in conditions characterized by excessive NO production and chronic inflammation. These findings will facilitate structure–activity relationship studies on the anti-inflammatory effects of procyanidins linked through methylene bridges compared with other types of procyanidins. Recent studies on the convenient condensation of natural products induced by cold plasma have supported this approach. Overall, this study provides a unique method for the semi-synthesis of procyanidins with significantly enhanced anti-inflammatory properties. Future research should explore the in vivo efficacy and safety profiles of these compounds to fully realize their therapeutic potential. Additionally, further investigations into the mechanistic pathways involved in their anti-inflammatory actions could provide deeper insights into their application as novel therapeutic agents for inflammation-related diseases.

## Figures and Tables

**Table 1:** ^1^H and ^13^C NMR shifts of new procyanidins **2**−**4**. ^1^.

		2		3		4	
Position		*δ*_H_ (*J* in Hz) ^2^	*δ*_C_, Type ^3^	*δ*_H_ (*J* in Hz) ^2^	*δ*_C_, Type ^3^	*δ*_H_ (*J* in Hz) ^2^	*δ*_C_, Type ^3^
C	2	4.71 (br s)	80.3, CH	4.95 (br s)	81.5, CH	4.78 (br s)	79.9, CH
	3	4.05 (m)	67.2, CH	4.15 (m)	67.1, CH	4.15 (m)	67.5, CH
	4	2.78 (dd, 16.8, 4.2) 2.62 (dd, 16.8, 3.0)	29.3, CH_2_	2.84 (dd, 16.2, 4.2) 2.74 (dd, 16.2, 3.0)	29.6, CH_2_	2.88 (dd, 16.8, 4.8) 2.75 (dd, 16.8, 3.0)	29.7, CH_2_
A	4a	―	100.6, C	―	102.1, C	―	101.6, C
	5	―	155.2, C	―	154.4, C	―	155.4, C
	6	―	106.5, C	5.96 (s)	96.6, CH	―	108.2, C
	7	―	155.8, C	―	151.4, C	―	153.7, C
	8	5.93 (s)	96.7, CH	―	106.3, C	6.04 (s)	96.0, CH
	8a	―	153.8, C	―	155.2, C	―	155.3, C
	9	3.79 (d, 15.6) 3.72 (d, 15.6)	17.1, CH_2_	3.79 (s)	17.9, CH_2_	3.74 (s)	17.8, CH_2_
B	1′	―	131.7, C	―	131.0, C	―	132.2, C
	2′	6.96 (br s)	115.4, CH	7.04 (d, 1.8)	115.6, CH	6.94 (d, 2.4)	115.3, CH
	3′	―	145.7, C	―	146.4, C	―	145.8, C
	4′	―	146.0, C	―	145.9, C	―	145.9, C
	5′	6.69 (d, 8.4)	116.0, CH	6.78 (d, 8.4)	116.1, CH	6.74 (d, 7.8)	115.9, CH
	6′	6.65 (dd, 8.4, 1.8)	119.7, CH	6.91 (dd, 8.4, 1.8)	120.1, CH	6.77 (dd, 7.8, 2.4)	119.4, CH
F	2	4.57 (br s)	80.1, CH	5.00 (br s)	81.7, CH	4.78 (br s)	79.9, CH
	3	4.10 (m)	66.9, CH	4.20 (m)	67.0, CH	4.15 (m)	67.5, CH
	4	2.68 (dd, 16.8, 4.2) 2.60 (dd, 16.8, 3.0)	29.2, CH_2_	2.90 (dd, 16.2, 4.2) 2.80 (dd, 16.2, 3.0)	29.5, CH_2_	2.88 (dd, 16.8, 4.8) 2.75 (dd, 16.8, 3.0)	29.7, CH_2_
D	4a	―	101.6, C	―	101.1, C	―	101.6, C
	5	―	151.8, C	―	155.4, C	―	155.4, C
	6	―	107.4, C	―	108.0, C	―	108.2, C
	7	―	153.3, C	―	155.3, C	―	153.7, C
	8	―	108.2, C	―	109.1, C	6.04 (s)	96.0, CH
	8a	―	152.0, C	―	151.3, C	―	155.3, C
	10	3.96 (d, 15.6) 3.89 (d, 15.6)	17.7, CH_2_	3.75 (d, 15.6) 3.71 (d, 15.6)	17.8, CH_2_	―	―
E	1′	―	131.9, C	―	130.9, C	―	132.2, C
	2′	6.86 (br s)	115.3, CH	7.10 (d, 1.8)	115.7, CH	6.94 (d, 2.4)	115.3, CH
	3′	―	145.8, C	―	146.2, C	―	145.8, C
	4′	―	146.1, C	―	145.8, C	―	145.9, C
	5′	6.71 (br s)	115.9, CH	6.82 (d, 8.4)	116.2, CH	6.74 (d, 7.8)	115.9, CH
	6′	6.72 (br s)	119.6, CH	6.87 (dd, 8.4, 1.8)	120.0, CH	6.77 (dd, 7.8, 2.4)	119.4, CH
I	2	4.91 (br s)	81.2, CH	4.75 (br s)	79.8, CH		
	3	4.19 (m)	66.8, CH	4.12 (m)	67.5, CH		
	4	2.86 (dd, 16.8, 4.2) 2.79 (dd, 16.8, 3.0)	29.1, CH_2_	2.78 (dd, 16.2, 4.8) 2.70 (dd, 16.2, 3.0)	29.7, CH_2_		
G	4a	―	100.7, C	―	101.5, C		
	5	―	154.9, C	―	153.8, C		
	6	6.03 (s)	97.2, CH	6.08 (s)	97.1, CH		
	7	―	153.0, C	―	153.3, C		
	8	―	106.7, C	―	107.5, C		
	8a	―	156.2, C	―	156.4, C		
H	1′	―	131.1, C	―	132.3, C		
	2′	7.05 (d, 1.8)	115.6, CH	6.92 (d, 1.8)	115.3, CH		
	3′	―	145.9, C	―	146.5, C		
	4′	―	146.2, C	―	146.1, C		
	5′	6.78 (d, 7.8)	116.1, CH	6.72 (d, 8.4)	115.9, CH		
	6′	6.85 (dd, 7.8, 1.8)	119.8, CH	6.75 (d, 8.4, 1.8)	119.4, CH		

^1^ Measurements were conducted in CD_3_OD, with chemical shift assignments derived from both 1D and 2D NMR spectroscopic analyses. Signals that overlapped were assigned using HSQC, HMBC, and ^1^H-^1^H COSY spectra without specifying the multiplicity. ^2^ The chemical shift data (*δ*) were obtained at 600 MHz. ^3^ The chemical shift data (*δ*) were obtained at 150 MHz.

## Data Availability

The data presented in this study are available upon request from the corresponding author.

## Supplementary Materials

The following supporting information can be downloaded at: https://www.mdpi.com/article/10.3390/ijms252011022/s1.
