# Supplementary material for: Inhibitory Effects of New Epicatechin Oligomers on Nitric Oxide Production
Source: Int J Mol Sci. 2024 Oct 14;25(20):11022. doi: 10.3390/ijms252011022 (PMC11507282; doi:10.3390/ijms252011022)
Supplement: Supplementary file 1 [file ijms-25-11022-s001.zip › ijms-3254092-supplementary.pdf]

Supporting Information

Inhibitory Effects of New Epicatechin Oligomers on Nitric Oxide Production

Gyeong Han Jeong [1,2,†], Hanui Lee [1,2,†], Byung Yeoup Chung [1,*], and Hyoung-Woo Bai [1,2,3,*]

[1] Research division for Biotechnology, Advanced Radiation Technology Institute (ARTI), Korea Atomic Energy Research Institute (KAERI), Jeongeup 56212, Republic of Korea
[2] Center for companion animal new drug development, Korea Institute of Toxicology (KIT), Jeongeup 56212, Republic of Korea
[3] Radiation Biotechnology and Applied Radioisotope Science, University of Science and Technology (UST), Daejeon 34113, Republic of Korea
* Correspondence: hbai@kaeri.re.kr (H.W.B.); bychung@kaeri.re.kr (B.Y.J.)
† These authors contributed equally to this work

Contents

Figure S1. Isolation procedure of oligomerized (−)-epicatechin reactant.

Figure S2. HPLC chromatograms of plasma treated (−)-epicatechin (**1**) and the isolated compounds **2**–**6**.

Figure S3. ^{1}H NMR spectrum of compound **2** in CD_3OD (600 MHz).

Figure S4. ^{13}C NMR spectrum of compound **2** in CD_3OD (150 MHz).

Figure S5. ^{1}H-^{1}H COSY spectrum of compound **2** in CD_3OD (600 MHz).

Figure S6. HSQC spectrum of compound **2** in CD_3OD (600 MHz).

Figure S7. HMBC spectrum of compound **2** in CD_3OD (600 MHz).

Figure S8. NOESY spectrum of compound **2** in CD_3OD (600 MHz).

Figure S9. HRFABMS spectrum of compound **2**.

Figure S10. ^{1}H NMR spectrum of compound **3** in CD_3OD (600 MHz).

Figure S11. ^{13}C NMR spectrum of compound **3** in CD_3OD (150 MHz).

Figure S12. ^{1}H-^{1}H COSY spectrum of compound **3** in CD_3OD (600 MHz).

Figure S13. HSQC spectrum of compound **3** in CD_3OD (600 MHz).

Figure S14. HMBC spectrum of compound **3** in CD_3OD (600 MHz).

Figure S15. HRFABMS spectrum of compound **3**.

Figure S16. ^{1}H NMR spectrum of compound **4** in CD_3OD (600 MHz).

Figure S17. ^{13}C NMR spectrum of compound **4** in CD_3OD (150 MHz).

Figure S18. ^{1}H-^{1}H COSY spectrum of compound **4** in CD_3OD (600 MHz).

Figure S19. HSQC spectrum of compound **4** in CD_3OD (600 MHz).

Figure S20. HMBC spectrum of compound **4** in CD_3OD (600 MHz).

Figure S21. NOESY spectrum of compound **4** in CD_3OD (600 MHz).

Figure S22. HRFABMS spectrum of compound **4**.

Figure S23. ^{1}H NMR spectrum of compound **5** in CD_3OD (600 MHz).

Figure S24. ^{13}C NMR spectrum of compound **5** in CD_3OD (150 MHz).

Figure S25. ^{1}H-^{1}H COSY spectrum of compound **5** in CD_3OD (600 MHz).

Figure S26. HSQC spectrum of compound **5** in CD_3OD (600 MHz).

Figure S27. HMBC spectrum of compound **5** in CD_3OD (600 MHz).

Figure S28. NOESY spectrum of compound **5** in CD_3OD (600 MHz).

Figure S29. FABMS spectrum of compound **5**.

Figure S30. ^{1}H NMR spectrum of compound **6** in CD_3OD (600 MHz).

Figure S31. ^{13}C NMR spectrum of compound **6** in CD_3OD (150 MHz).

Figure S32. ^{1}H-^{1}H COSY spectrum of compound **6** in CD_3OD (600 MHz).

Figure S33. HSQC spectrum of compound **6** in CD_3OD (600 MHz).

Figure S34. HMBC spectrum of compound **6** in CD_3OD (600 MHz).

Figure S35. NOESY spectrum of compound **6** in CD_3OD (600 MHz).

Figure S36. FABMS spectrum of compound **6**.

Figure S37. CD spectra of new compounds **2**–**4**.

Figure S38. Chemical structures of the isolated compounds **2–6**.

Figure S39. Western blotting data for iNOS and GAPDH of compounds **2** and **3** in LPS-stimulated RAW264.7 cells.

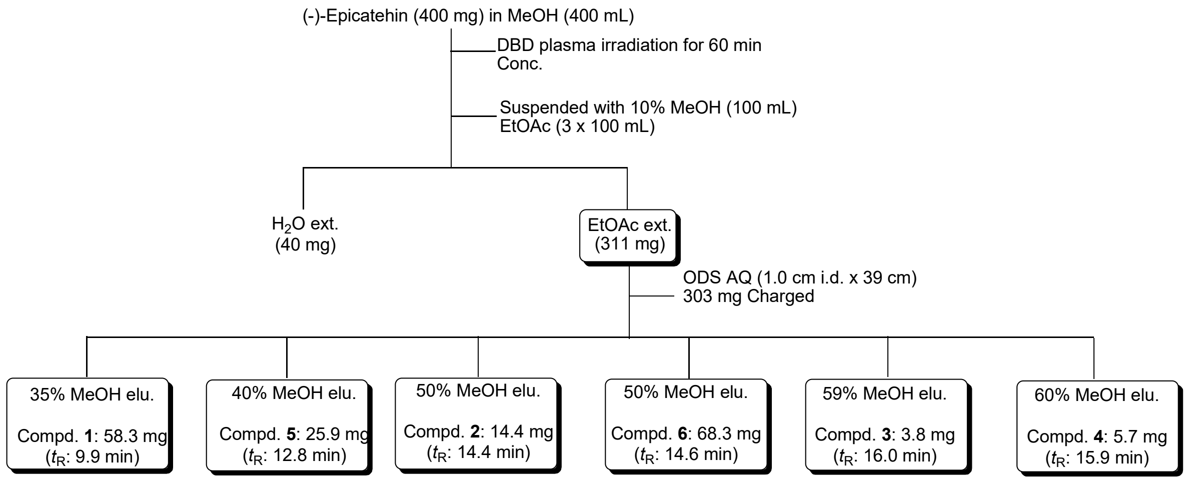

Figure S1. Isolation procedure of oligomerized (−)-epicatechin reactant.

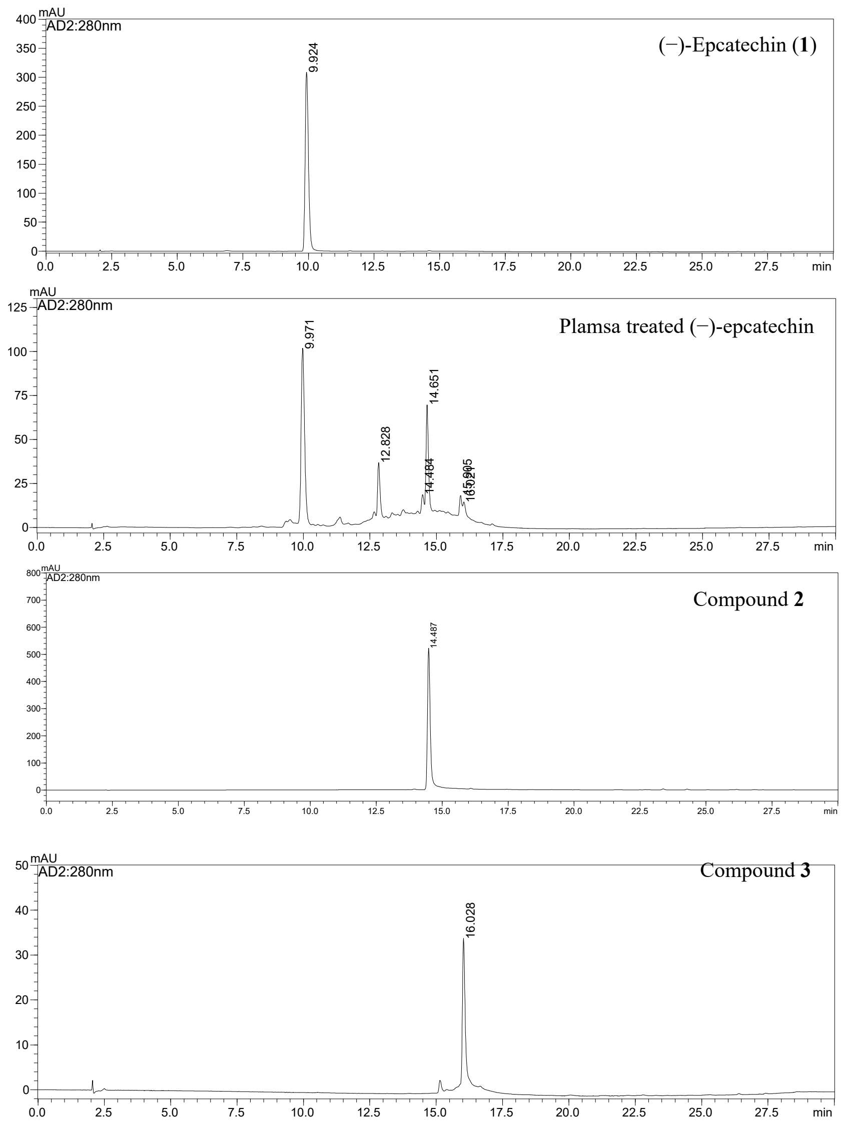

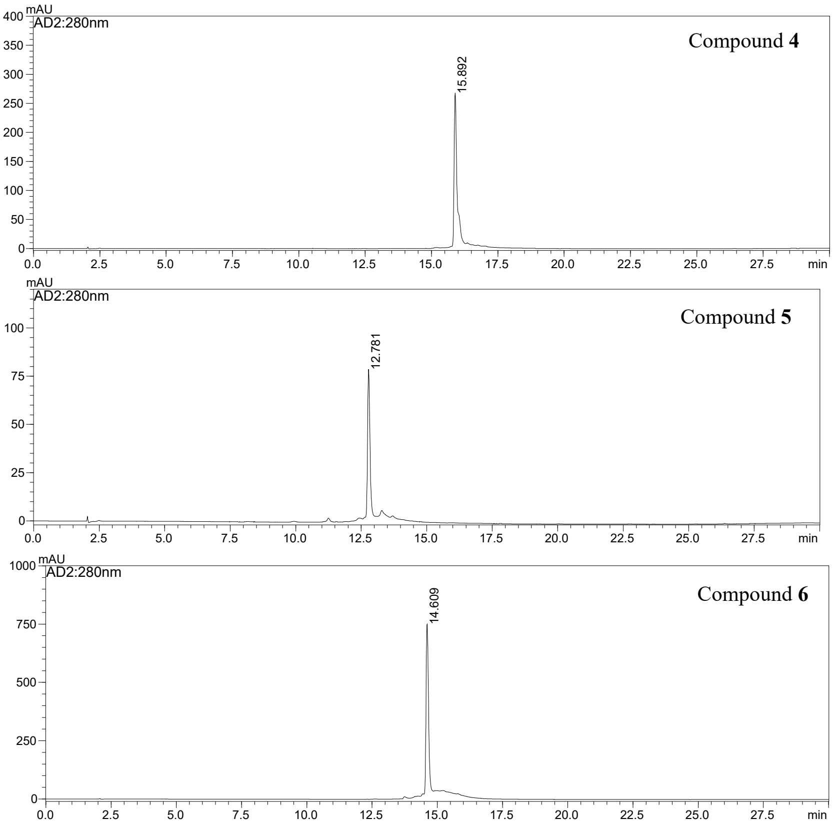

Figure S2. HPLC chromatograms of plasma treated (−)-epicatechin (**1**) and the isolated compounds **2**–**6**.

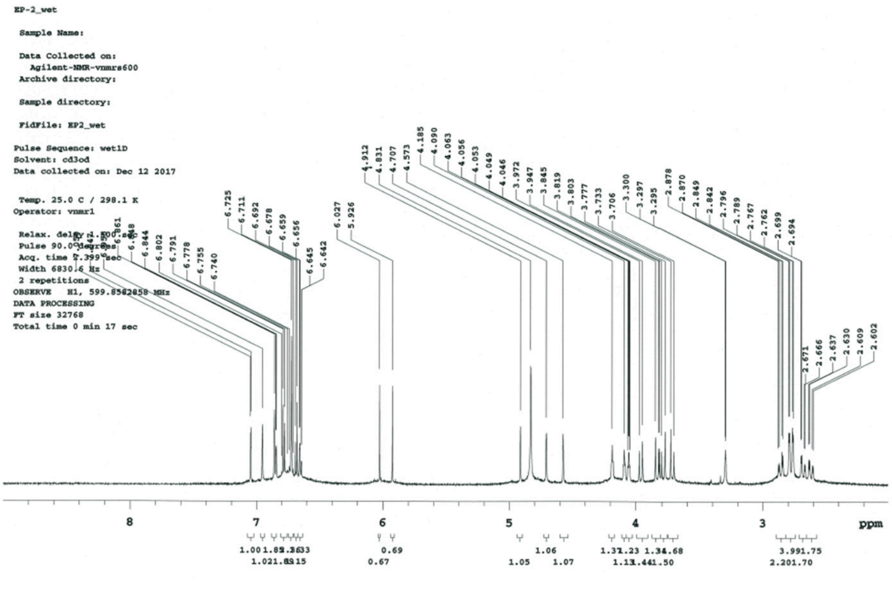

Figure S3. ^{1}H NMR spectrum of compound **2** in CD_3OD (600 MHz).

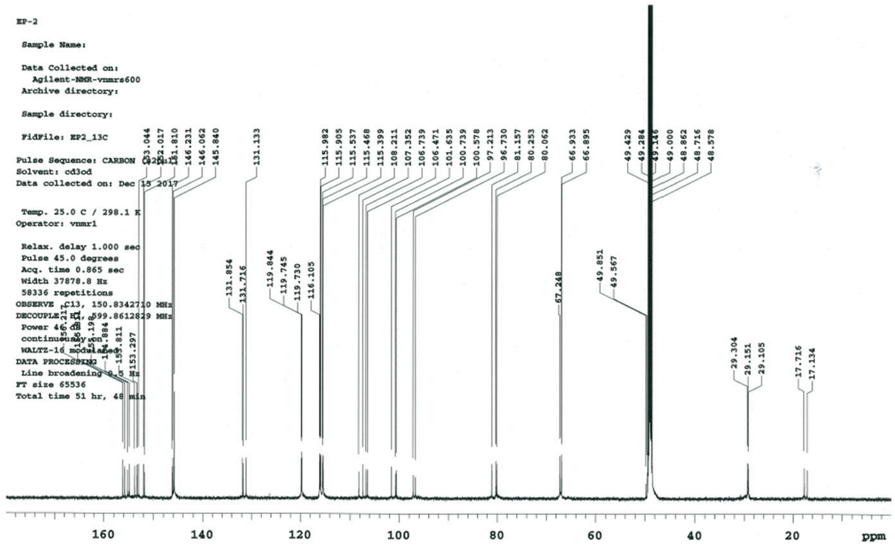

Figure S4. ^{13}C NMR spectrum of compound **2** in CD_3OD (150 MHz).

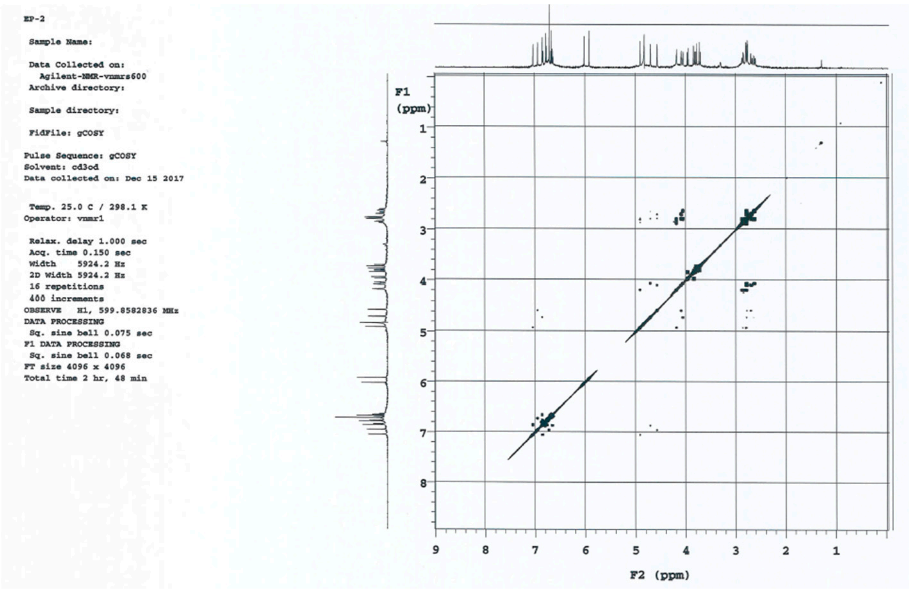

Figure S5. ^{1}H-^{1}H COSY spectrum of compound **2** in CD_3OD (600 MHz).

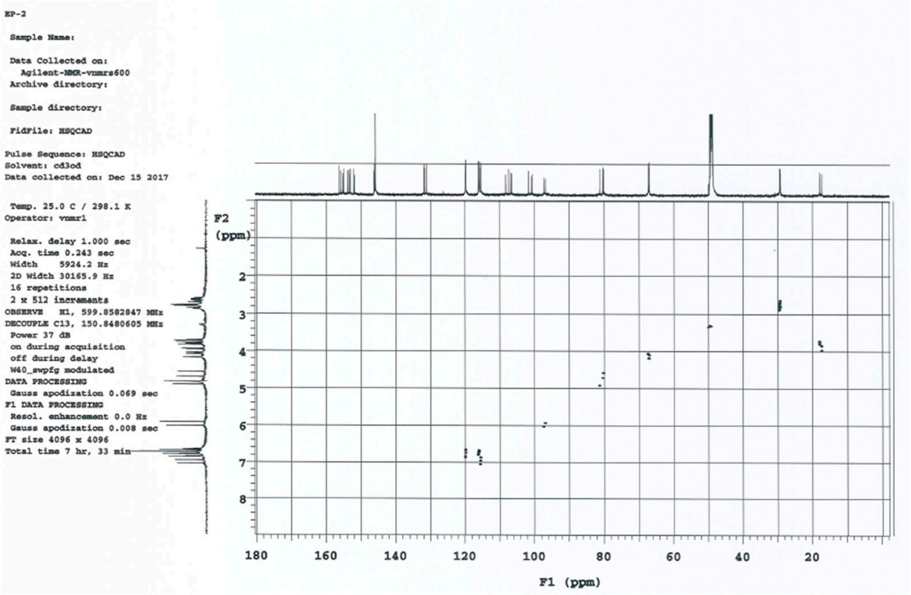

Figure S6. HSQC spectrum of compound **2** in CD_3OD (600 MHz).

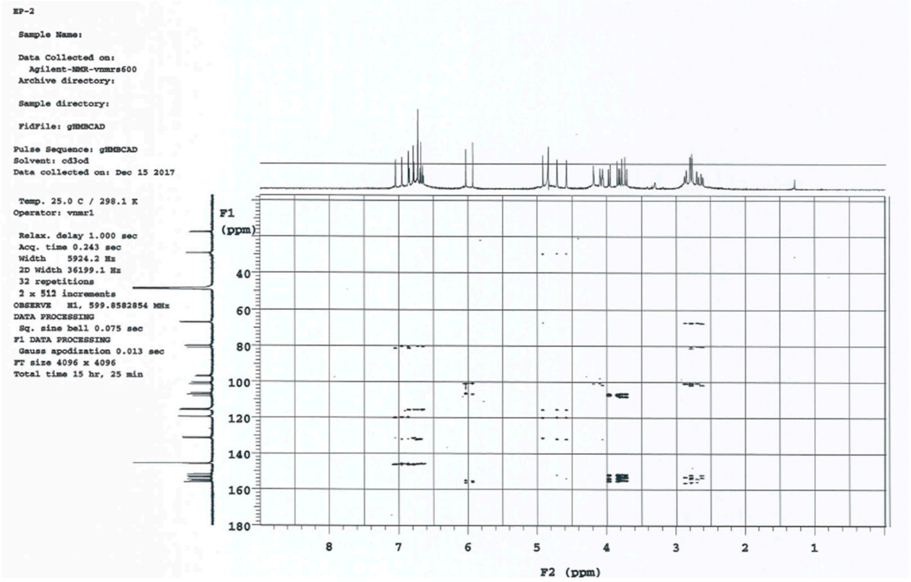

Figure S7. HMBC spectrum of compound **2** in CD_3OD (600 MHz).

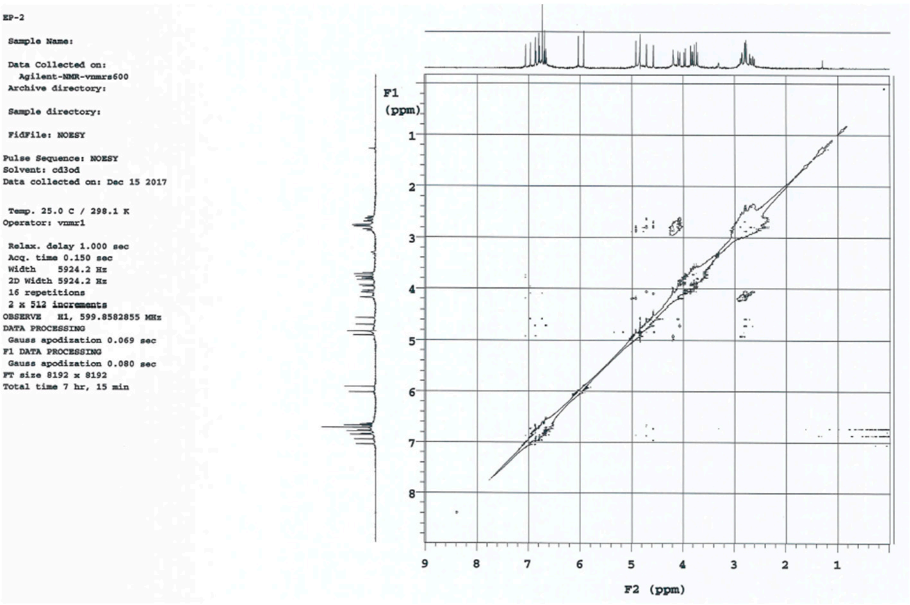

Figure S8. NOESY spectrum of compound **2** in CD_3OD (600 MHz).

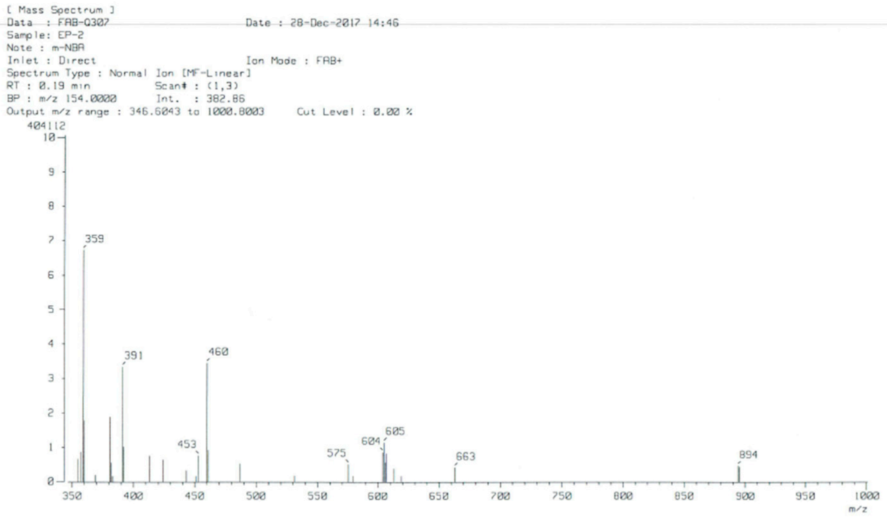

[Elemental Composition]
Data : FAB-Q326
Date : 29-Dec-2017 17:46
Page: 1
Sample: EP-2
Note : m-NBA
Inlet : Direct
Ion Mode : FAB+
RT : 0.76 min
Scan#: (19,53)
Elements : C 100/0, H 100/0, O 20/15
Mass Tolerance : 20ppm, 5mmu if m/z < 250, 10mmu if m/z > 500
Unsaturation (U.S.) : -0.5 - 100.0

Observed m/z	Int%	Err[ppm / mmu]	U.S.	Composition
894.2359	7.0	-1.4 / -1.2	27.0	C 47 H 42 O 18

```
[ Theoretical Ion Distribution ]                                    Page: 1
Molecular Formula : C47 H42 O18
                        (m/z   894.2371, MW   894.8397, U.S. 27.0)
Base Peak :   894.2371, Averaged MW :   894.8349(a),   894.8356(w)

    m/z       INT.
  894.2371 100.0000 ***************************************************
  895.2405  52.9607 ***************************
  896.2433  17.3419 **********
  897.2461   4.2336 **
  898.2488   0.8449
  899.2514   0.1442
  900.2540   0.0217
  901.2565   0.0029
  902.2590   0.0004
```

Figure S9. HRFABMS spectrum of compound **2**.

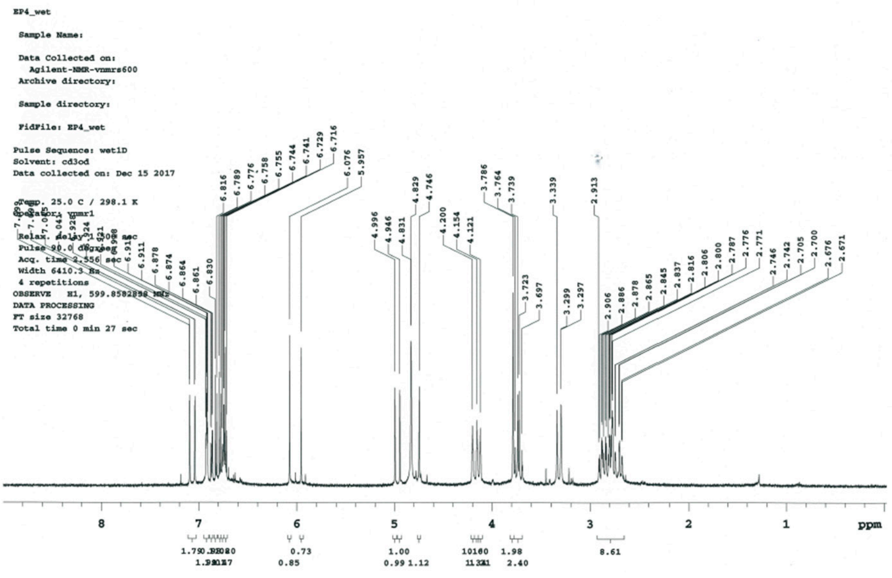

Figure S10. ^{1}H NMR spectrum of compound **3** in CD_3OD (600 MHz).

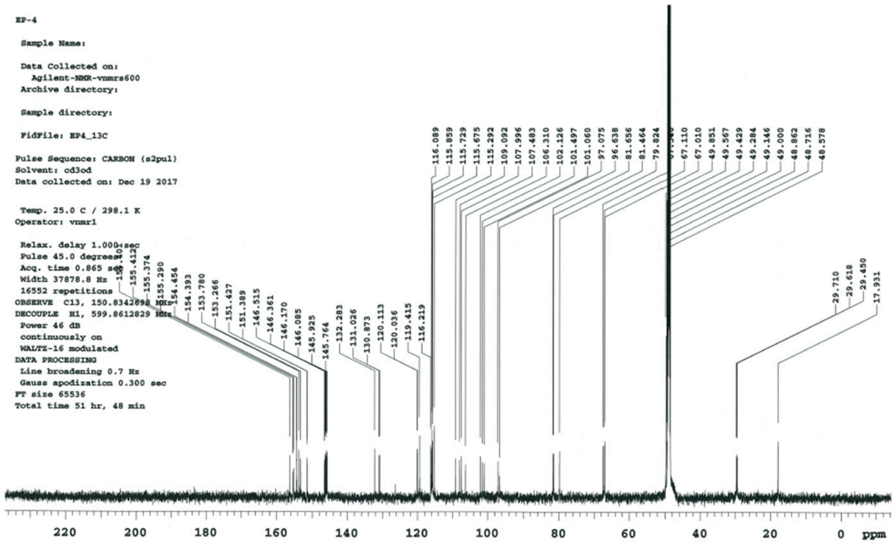

Figure S11. ^{13}C NMR spectrum of compound **3** in CD_3OD (150 MHz).

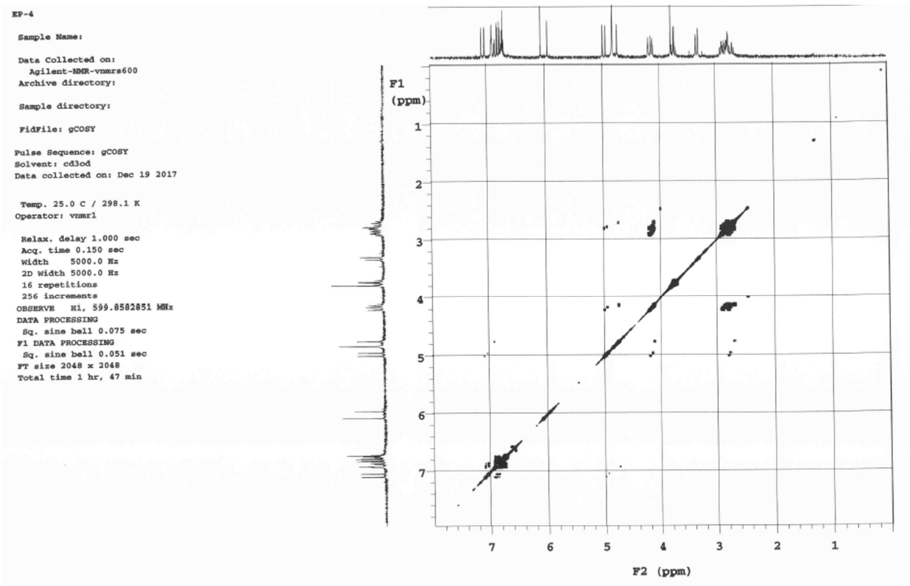

Figure S12. ^1H-^1H COSY spectrum of compound **3** in CD_3OD (600 MHz).

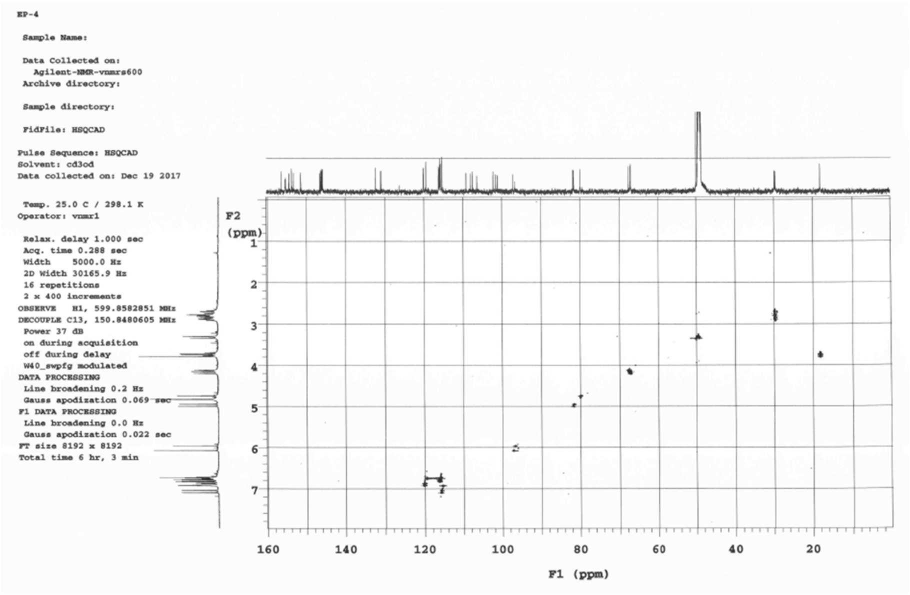

Figure S13. HSQC spectrum of compound **3** in CD_3OD (600 MHz).

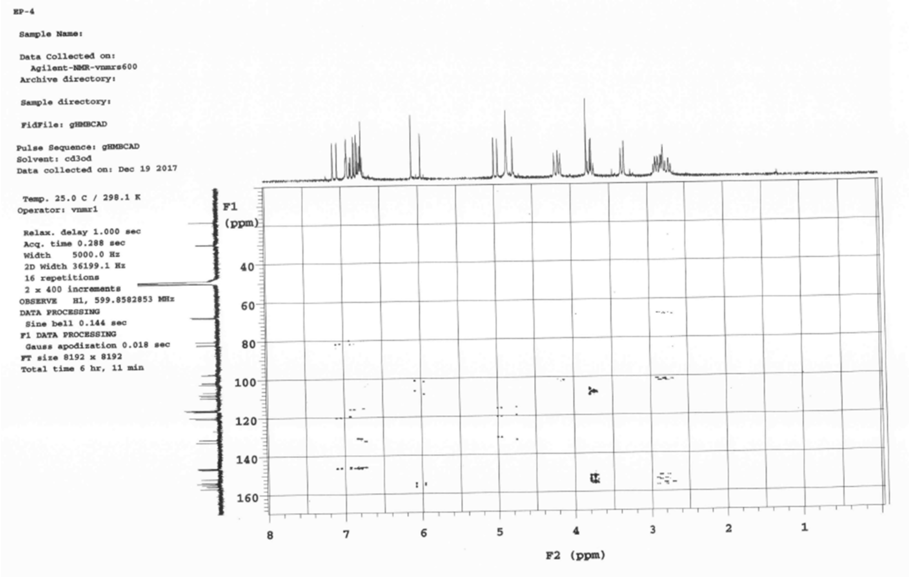

Figure S14. HMBC spectrum of compound **3** in CD_3OD (600 MHz).

```
[ Mass Spectrum ]
Data  : FAB-Q309                         Date : 28-Dec-2017 14:53
Sample: EP-4
Note : m-NBA
Inlet : Direct                           Ion Mode : FAB+
Spectrum Type : Normal Ion [MF-Linear]
RT : 0.00 min          Scan# : (1,2)
BP : m/z 154.0000      Int.   : 247.98
Output m/z range : 11.4679 to 1000.8002      Cut Level : 0.00 %
```

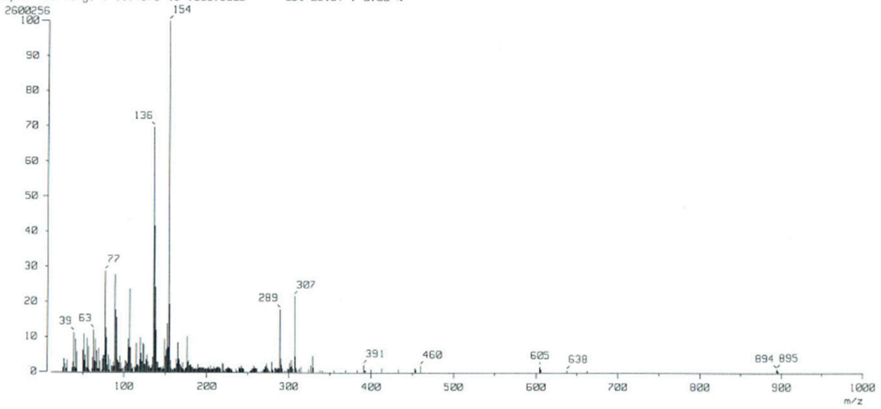


```
[ Elemental Composition ]                                    Page: 1
Data   : FAB-Q327                  Date : 02-Jan-2018 16:08
Sample: EP-4
Note : m-NBA
Inlet : Direct                     Ion Mode : FAB+
RT : 2.13 min                      Scan#: (72,126)
Elements : C 100/0, H 100/0, O 20/15
Mass Tolerance       : 20ppm, 5mmu if m/z < 250, 10mmu if m/z > 500
Unsaturation (U.S.) : -0.5 - 100.0

Observed m/z Int%   Err[ppm / mmu]    U.S.  Composition
  894.2372     8.5     +0.1 /  +0.1   27.0  C 47 H 42 O 18
```

```
[ Theoretical Ion Distribution ]                             Page: 1
Molecular Formula : C47 H42 O18
                      (m/z   894.2371, MW   894.8397, U.S. 27.0)
Base Peak :     894.2371, Averaged MW :   894.8349(a),   894.8356(w)

    m/z        INT.
  894.2371 100.0000 ****************************************************************
  895.2405  52.9607 ********************************
  896.2433  17.3419 **********
  897.2461   4.2336 **
  898.2488   0.8449
  899.2514   0.1442
  900.2540   0.0217
  901.2565   0.0029
  902.2590   0.0004
```

Figure S15. HRFABMS spectrum of compound **3**.

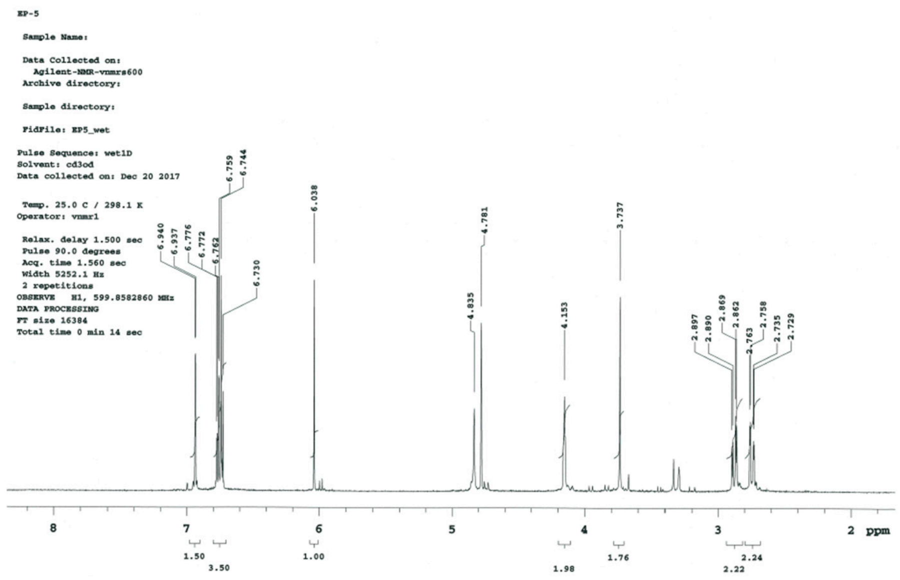

Figure S16. ^{1}H NMR spectrum of compound **4** in CD_3OD (600 MHz).

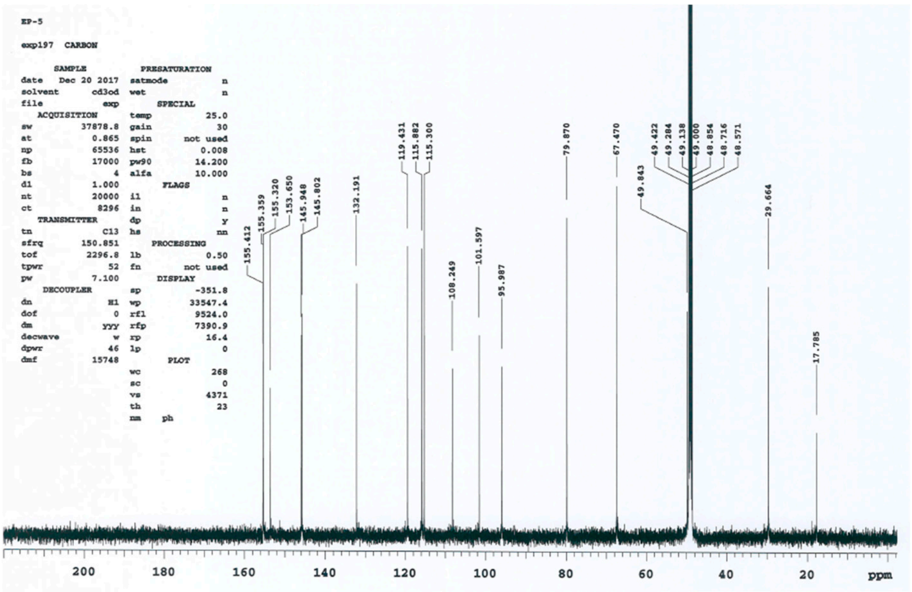

Figure S17. ^{13}C NMR spectrum of compound **4** in CD_3OD (150 MHz).

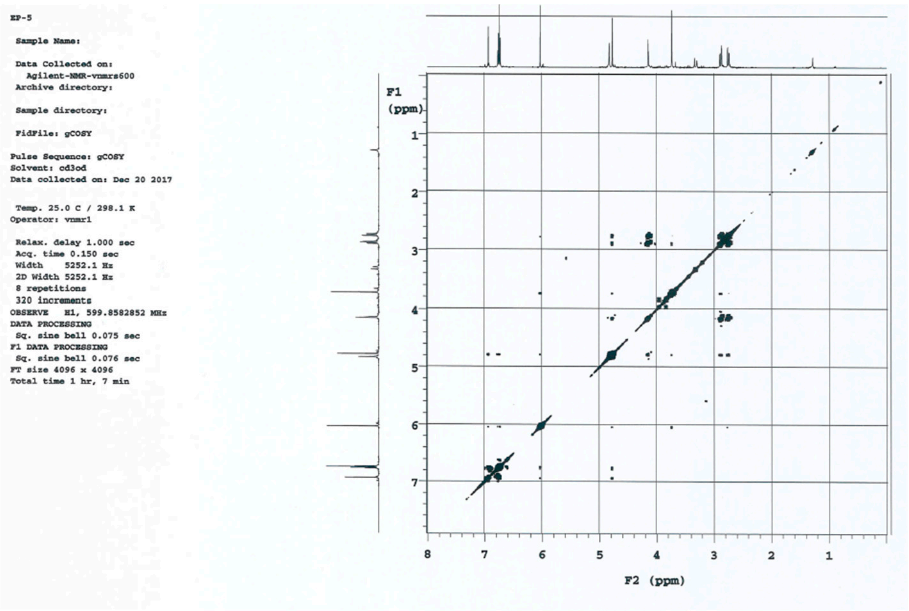

Figure S18. ^{1}H-^{1}H COSY spectrum of compound **4** in CD_3OD (600 MHz).

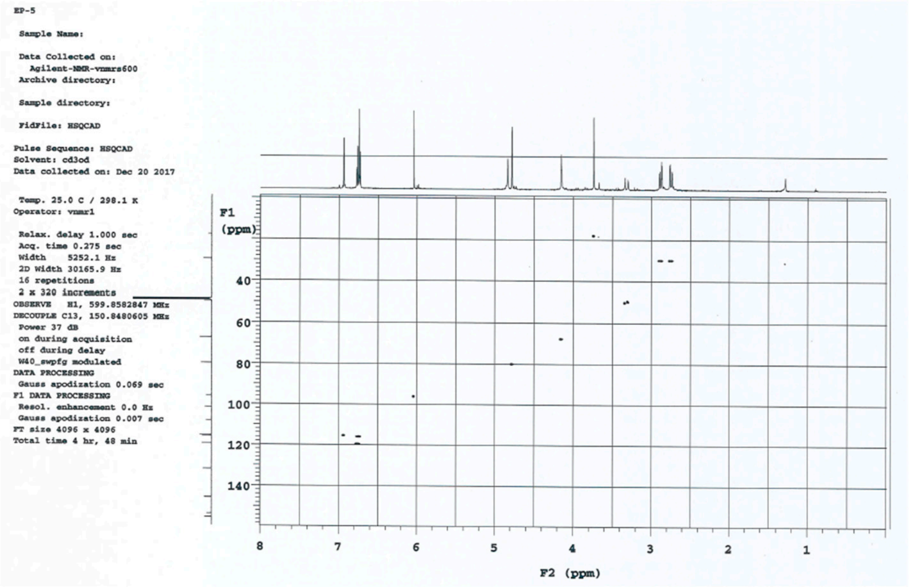

Figure S19. HSQC spectrum of compound **4** in CD_3OD (600 MHz).

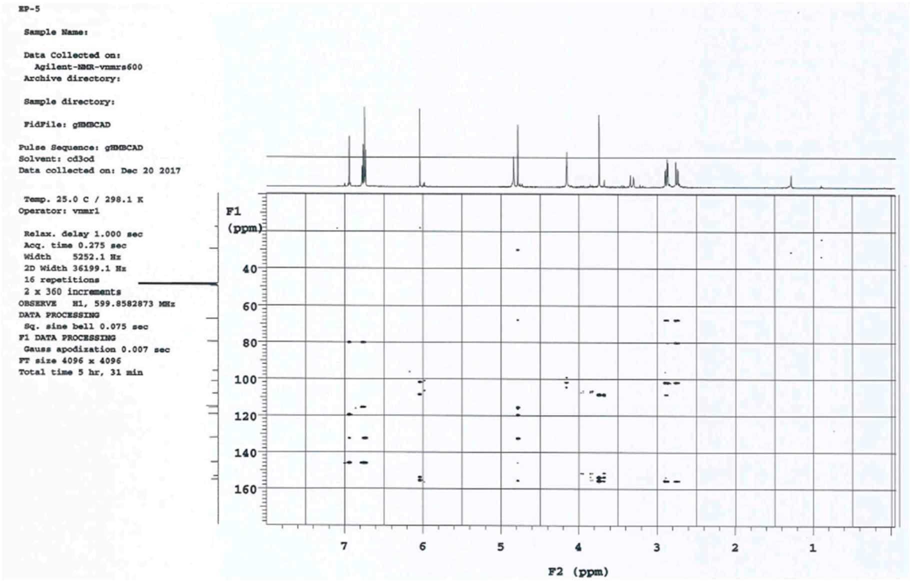

Figure S20. HMBC spectrum of compound **4** in CD_3OD (600 MHz).

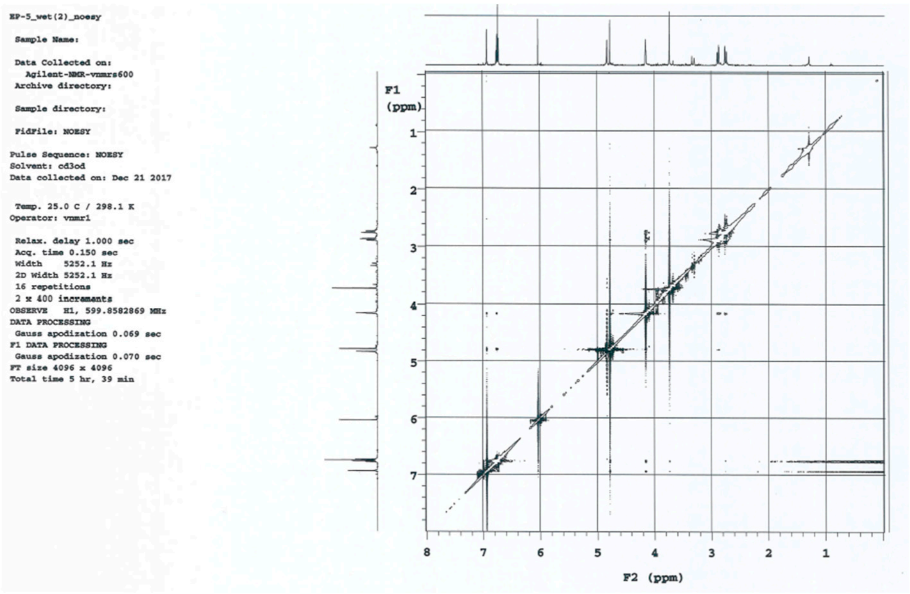

Figure S21. NOESY spectrum of compound **4** in CD_3OD (600 MHz).

```
[ Mass Spectrum ]
Data   : FAB-Q308                          Date : 28-Dec-2017 14:50
Sample: EP-5
Note  : m-NBA
Inlet : Direct                             Ion Mode : FAB+
Spectrum Type : Normal Ion [MF-Linear]
RT : 1.65 min            Scan# : (9,12)
BP : m/z 154.0000        Int.  : 97.06
Output m/z range : 10.0000 to 650.1729      Cut Level : 0.00 %
```

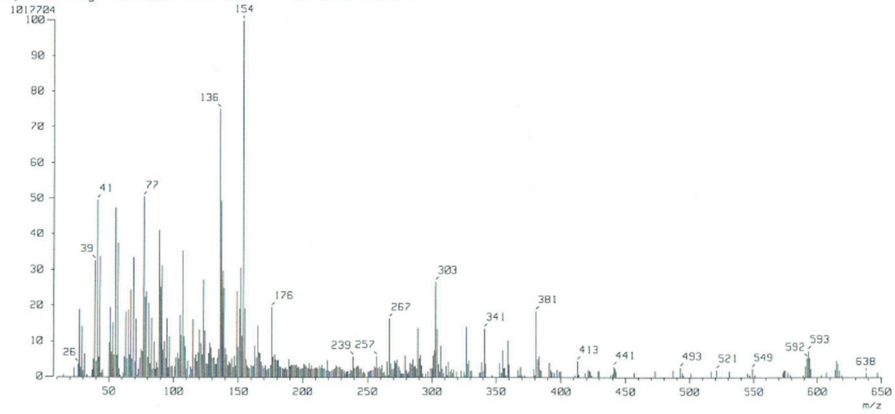


```
[ Elemental Composition ]                                        Page: 1
Data   : FAB-Q324                   Date : 29-Dec-2017 17:01
Sample: EP-5
Note  : m-NBA
Inlet : Direct                      Ion Mode : FAB+
RT : 0.66 min                       Scan#: (25,38)
Elements : C 100/0, H 100/0, O 15/10
Mass Tolerance      : 20ppm, 5mmu if m/z < 250, 10mmu if m/z > 500
Unsaturation (U.S.) : -0.5 - 100.0

Observed m/z Int%   Err[ppm / mmu]   U.S.  Composition
  592.1584    12.5     +0.6 /  +0.4   18.0  C 31 H 28 O 12
```

```
[ Theoretical Ion Distribution ]                                 Page: 1
Molecular Formula : C31 H28 O12
                         (m/z   592.1581, MW   592.5561, U.S. 18.0)
Base Peak :    592.1581, Averaged MW :   592.5529(a),    592.5536(w)

    m/z        INT.
  592.1581 100.0000 ***********************************************************
  593.1614  34.9364 ********************
  594.1641   8.3166 *****
  595.1668   1.4847 *
  596.1693   0.2194
  597.1719   0.0278
  598.1744   0.0031
  599.1769   0.0003
```

Figure S22. HRFABMS spectrum of compound **4**.

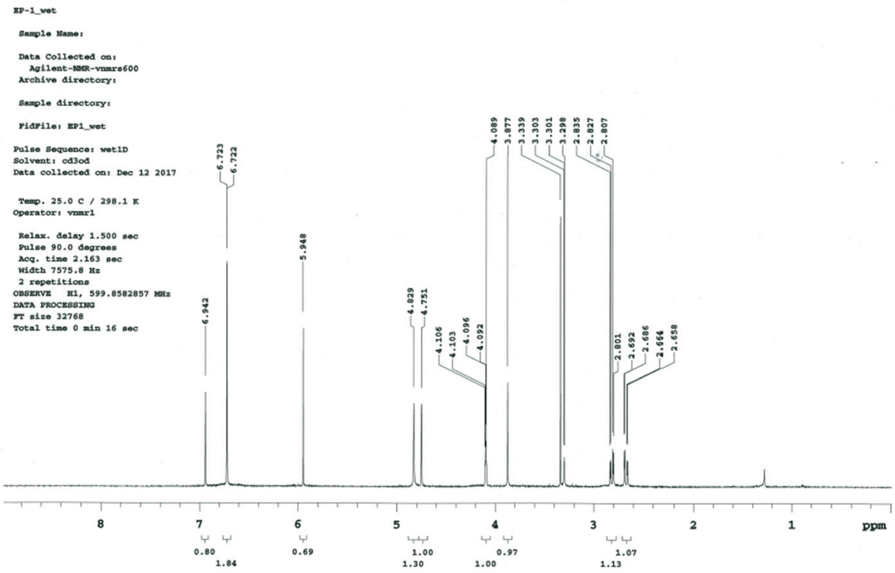

Figure S23. ^{1}H NMR spectrum of compound **5** in CD_3OD (600 MHz).

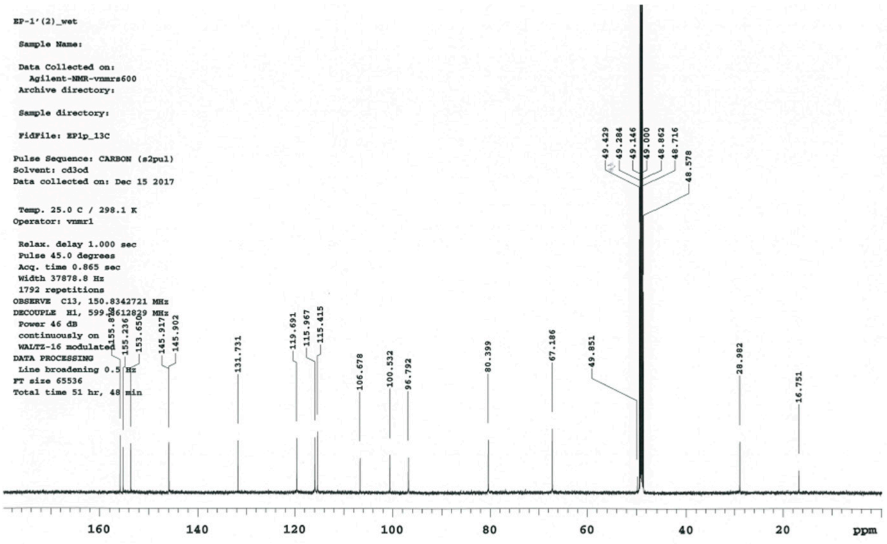

Figure S24. ^{13}C NMR spectrum of compound **5** in CD_3OD (150 MHz).

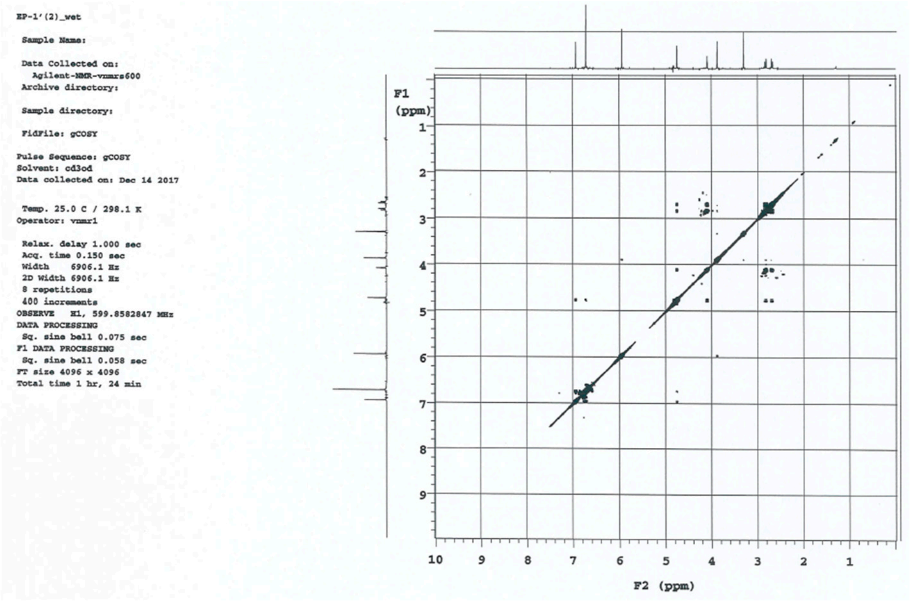

Figure S25. ^{1}H-^{1}H COSY spectrum of compound **5** in CD_3OD (600 MHz).

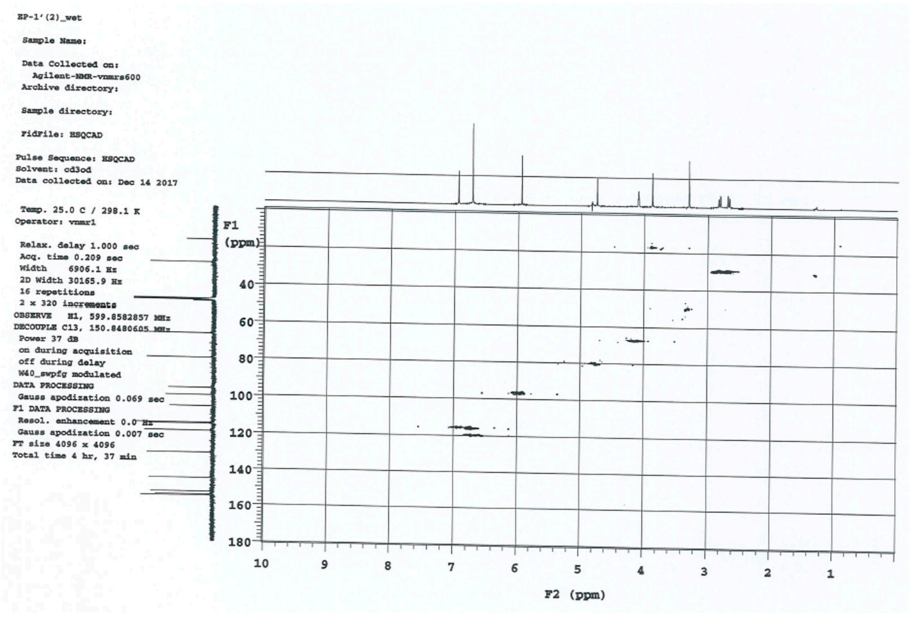

Figure S26. HSQC spectrum of compound **5** in CD_3OD (600 MHz).

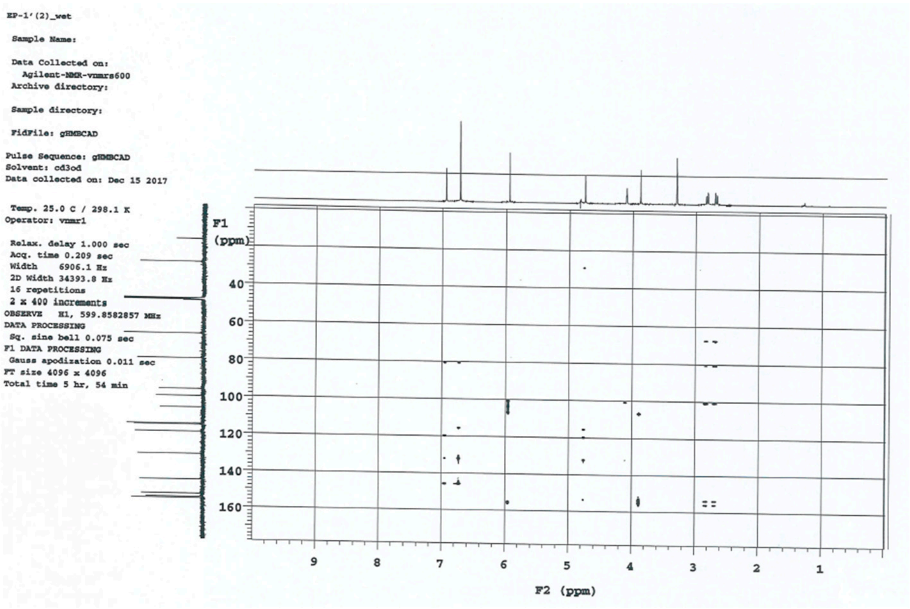

Figure S27. HMBC spectrum of compound **5** in CD_3OD (600 MHz).

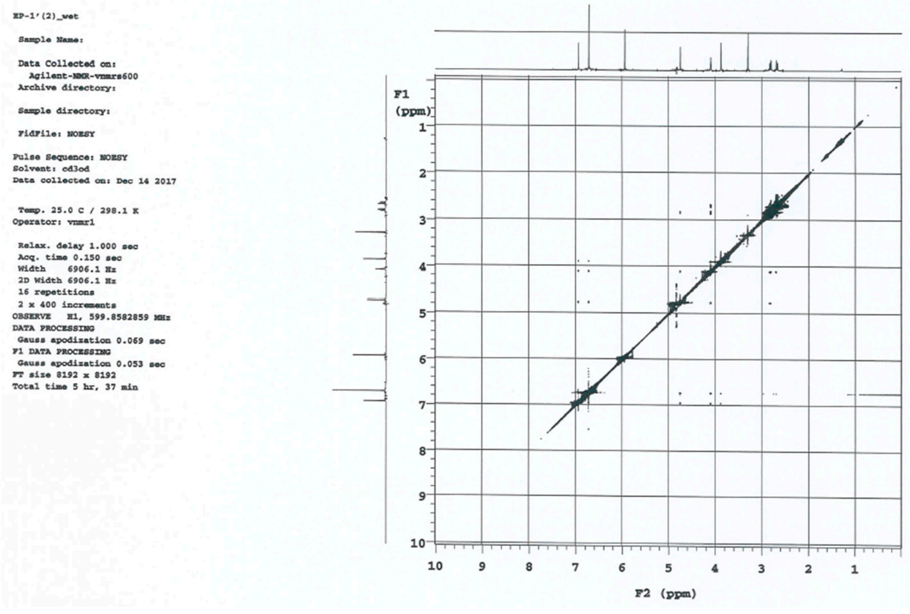

Figure S28. NOESY spectrum of compound **5** in CD_3OD (600 MHz).

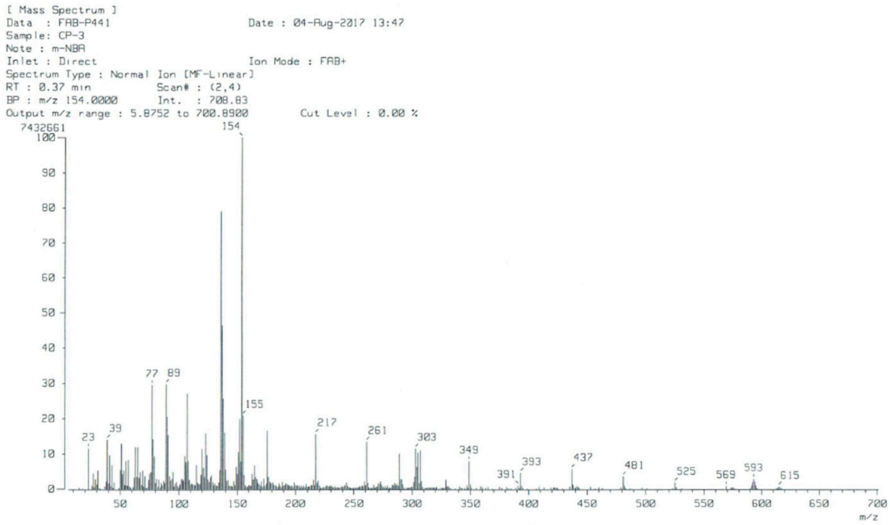

Figure S29. FABMS spectrum of compound **5**.

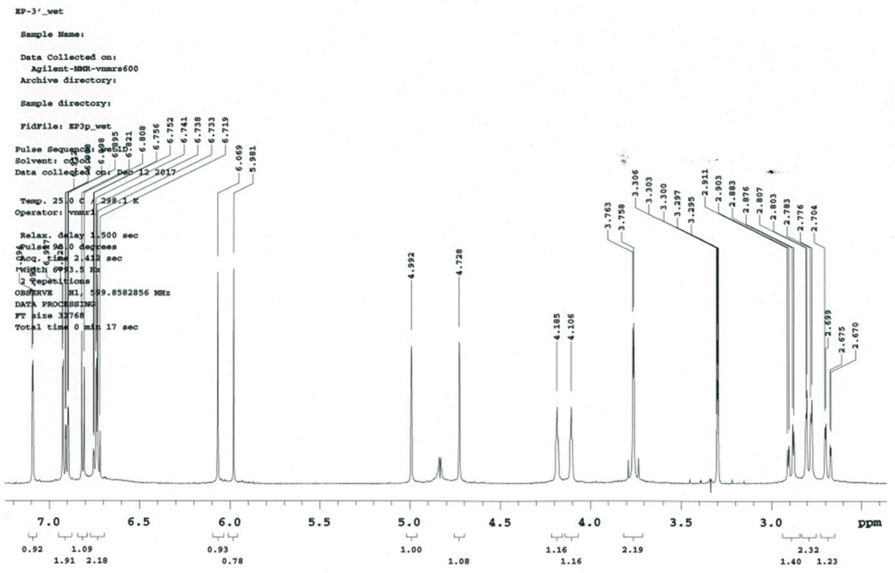

Figure S30. ^{1}H NMR spectrum of compound **6** in CD_3OD (600 MHz).

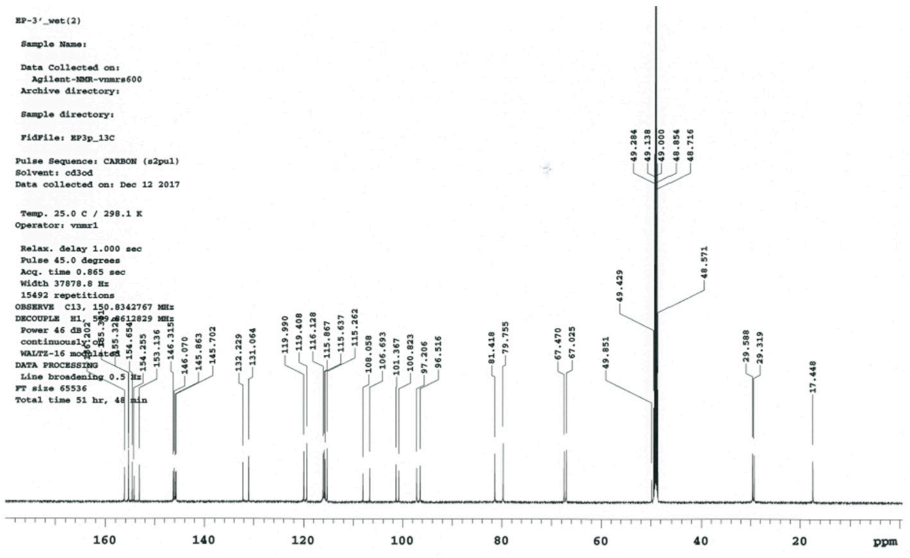

Figure S31. ^{13}C NMR spectrum of compound **6** in CD_3OD (150 MHz).

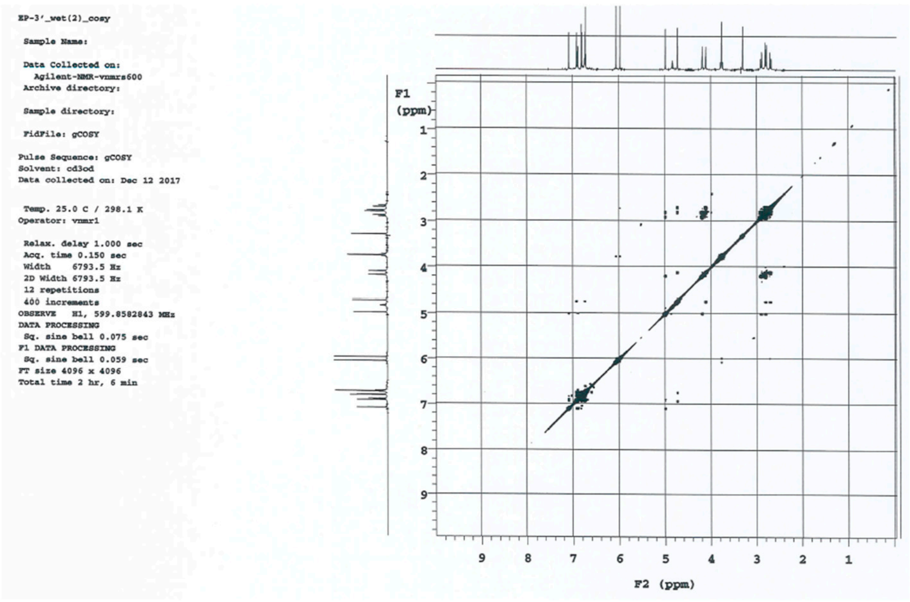

Figure S32. ^{1}H-^{1}H COSY spectrum of compound **6** in CD_3OD (600 MHz).

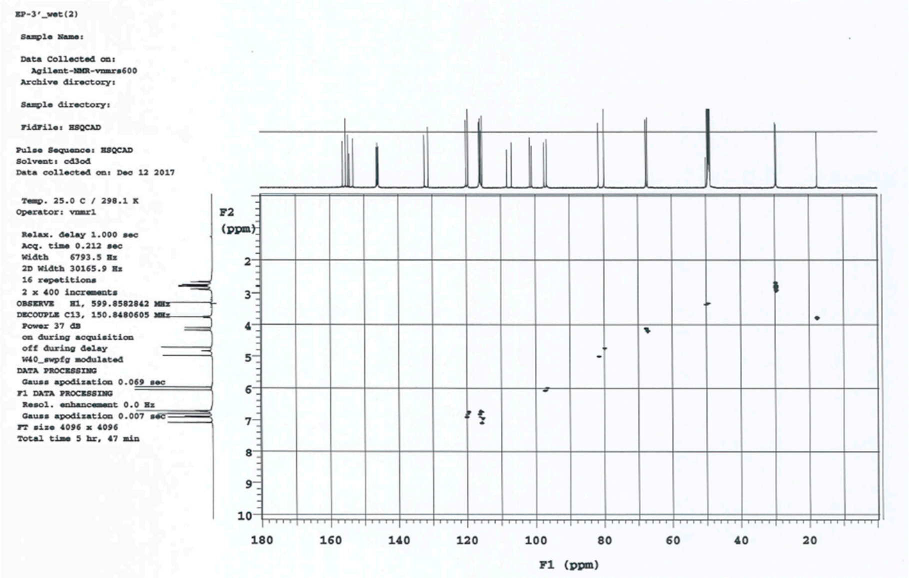

Figure S33. HSQC spectrum of compound **6** in CD_3OD (600 MHz).

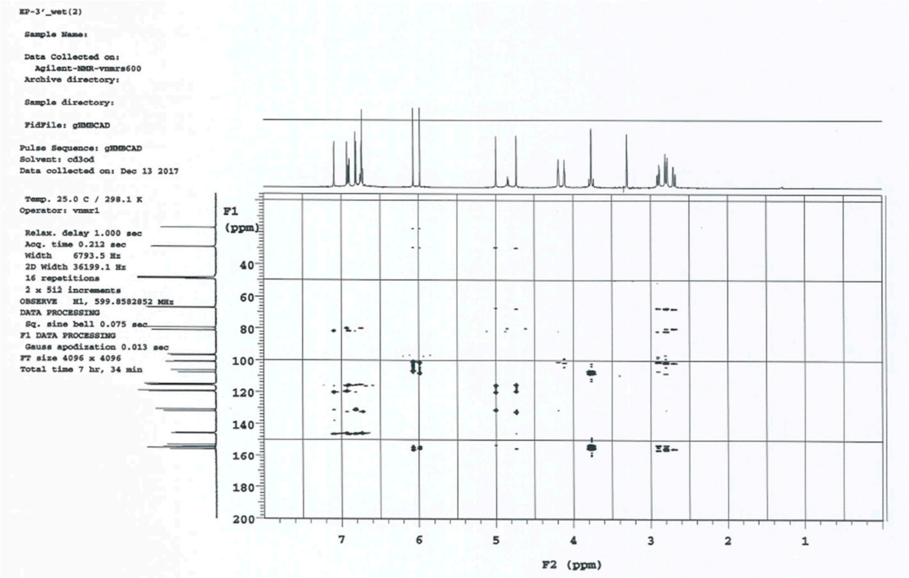

Figure S34. HMBC spectrum of compound **6** in CD_3OD (600 MHz).

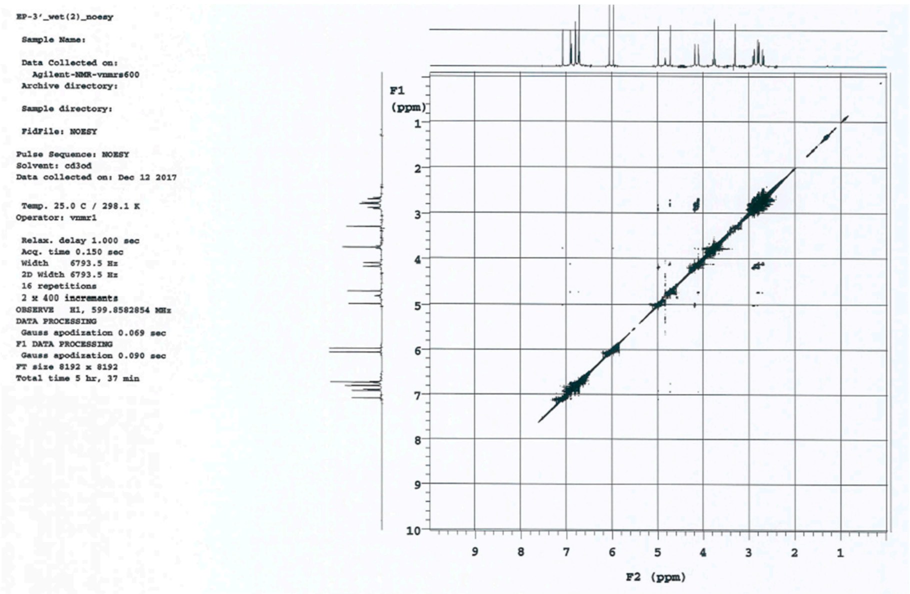

Figure S35. NOESY spectrum of compound **6** in CD_3OD (600 MHz).

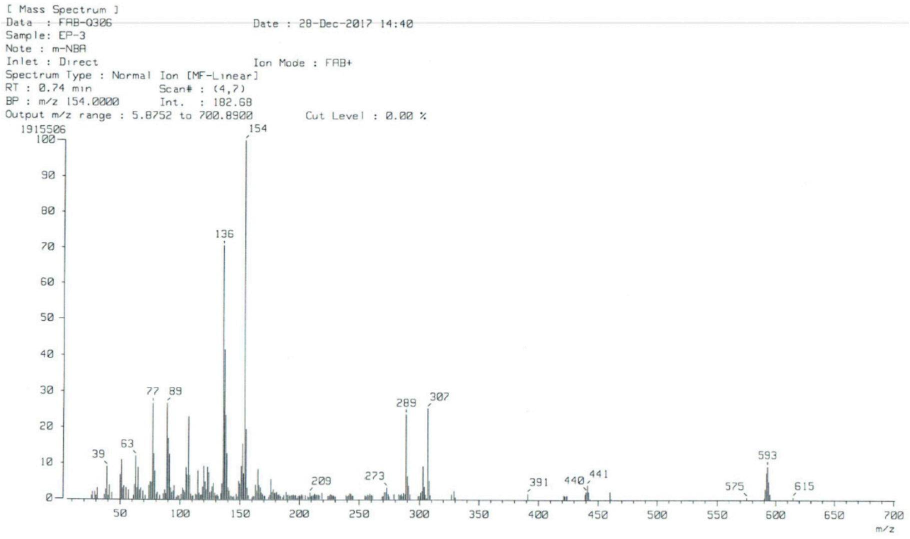

Figure S36. FABMS spectrum of compound **6**.

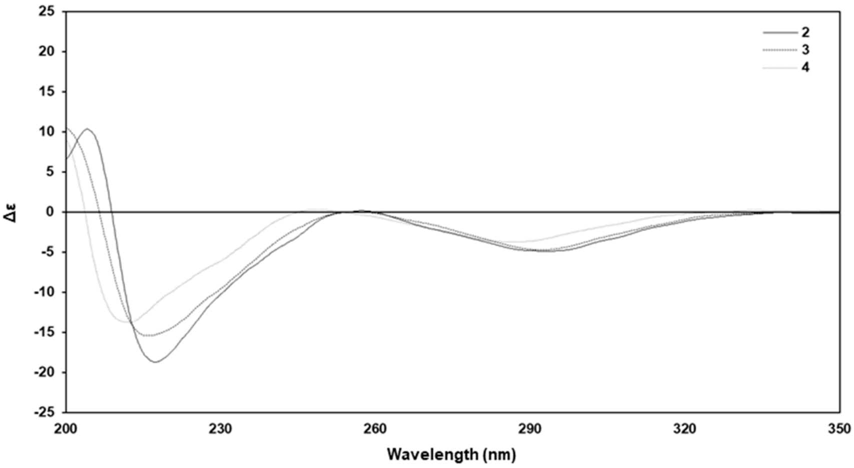

Figure S37. CD spectra of new compounds **2**–**4**.

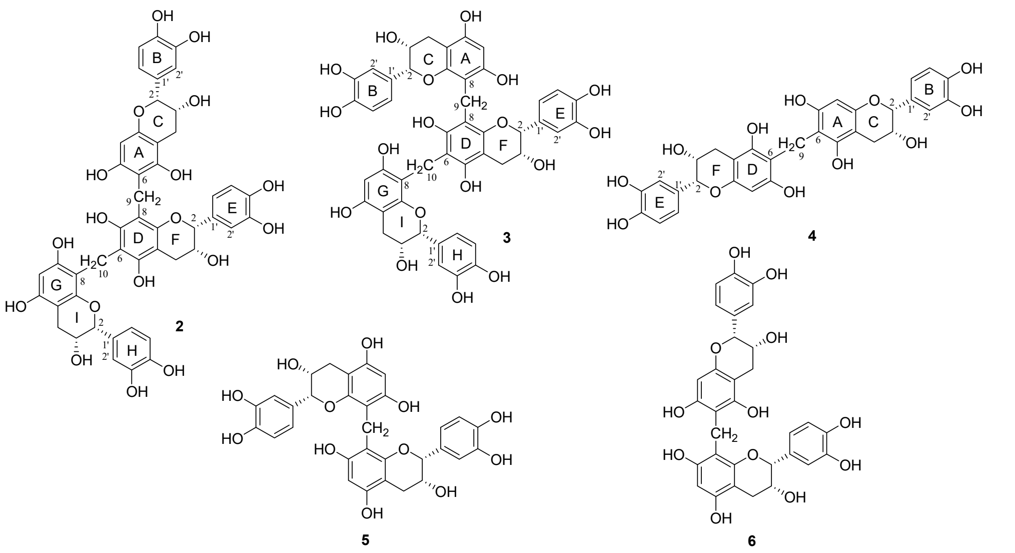

Figure S38. Chemical structures of the isolated compounds **2**–**6**.

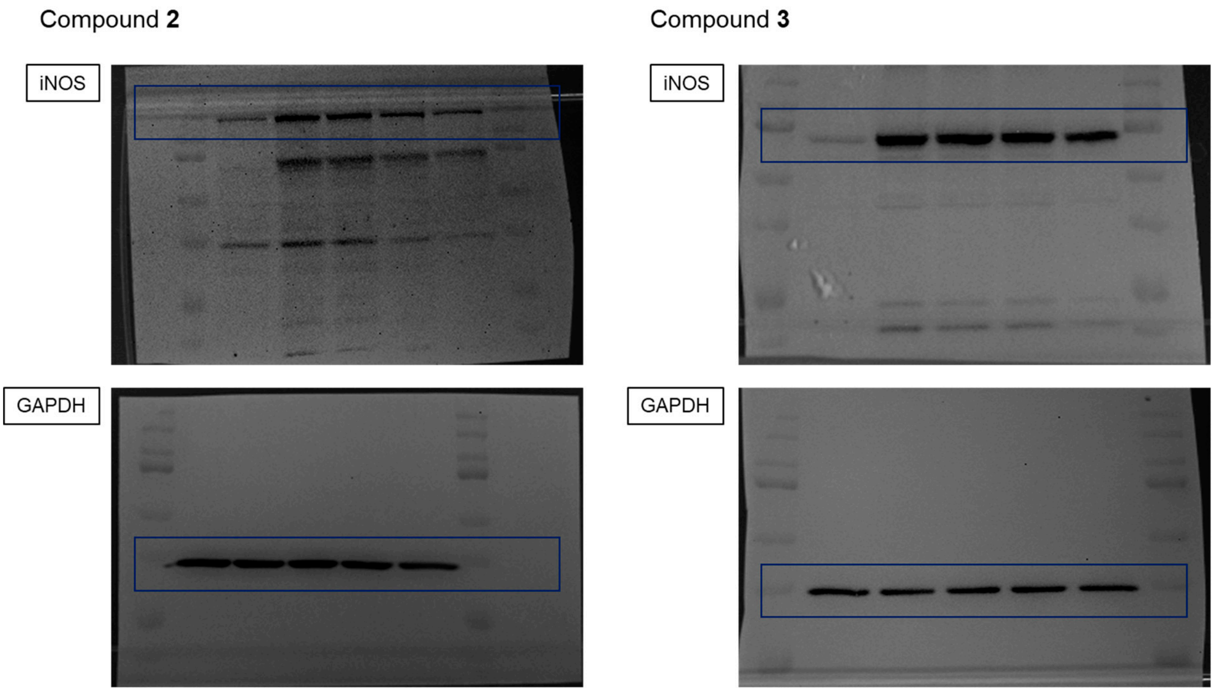

Figure S39. Western blotting data for iNOS and GAPDH of compounds **2** and **3** in LPS-stimulated RAW264.7 cells.
